# Genetic diversity and mobile genetic element associated multidrug resistance in *Salmonella enterica* from broiler chickens in Egypt

**DOI:** 10.1038/s41598-026-59913-w

**Published:** 2026-07-03

**Authors:** Mona Salem, Basel A. Abukhadra, Reem Ghabbour, Marwa El-Metwaly El-Metwaly, Gamal Younis, Amal Awad

**Affiliations:** 1 Department of Bacteriology, Mycology and Immunology, Faculty of Veterinary Medicine, Mansoura University, Mansoura, 35516 Egypt; 2 Department of Virology, Faculty of Veterinary Medicine, Mansoura University, 35516 El Gomhoria Street, Mansoura, Egypt; 3 Gastro-Enterology Surgery Center (GEC), Mansoura University, Mansoura, Egypt; 4 Department of Preclinical Veterinary Sciences, Faculty of Veterinary Medicine, Delta University for Science and Technology, Gamasa, Egypt

**Keywords:** *Salmonella enterica*, Broiler chicken, Integron, Antimicrobial resistance, ERIC-PCR, Public health concern, Egypt, Genetics, Microbiology, Molecular biology

## Abstract

*Salmonella enterica* remains a leading foodborne zoonotic pathogen worldwide, with poultry serving as a major reservoir and vehicle for antimicrobial resistance dissemination to humans. This study investigated the genotypic basis and distribution of multidrug resistance (MDR) among 29 *S. enterica* isolates from broiler farms in Egypt, emphasizing the role of mobile genetic elements as integrons and the assessment of genetic relatedness using ERIC-PCR. Molecular screening revealed high prevalence of resistance determinants, including *flo*R (93.1%), *tet*A (86.2%), *aph*A1 (82.8%), *cml*A (75.9%), *ere*A (75.9%), *sul*I (62.1%), *aad*A1 (51.7%), *dfr*A1 (48.3%), *aac*(3)-IV (44.8%), *tet*B (41.4%), *sul*II (31.0%), *aac*(6′)-Ib-cr (24.1%), *cat*A1 (20.7%), *fos*A3 (20.7%), and *qnr*A (10.3%). High-risk serovars, including *S.* Jerusalem, *S.* Colorado, and *S.* Kentucky, harbored multiple resistance genes and exhibited pronounced XDR profiles. Notably, this study reports the detection of *aph*A1 and *fos*A3 in *Salmonella* isolates derived from broiler chickens, which may represent an early or uncommon finding in Egypt. Many resistance genes were associated with horizontally transferable class 1 integron, underscoring its key role in the dissemination of multidrug resistance (MDR) within poultry systems and along the food chain. ERIC-PCR genotyping segregated isolates into two major genetic groups with seven sub-clusters, reflecting clustering patterns and genetic diversity among the isolates, alongside notable heterogeneity in resistance, virulence, and biofilm-associated genes.Overall, poultry in Egypt represents a significant reservoir of genetically diverse and potentially transmissible MDR *S. enterica*, highlighting the need for enhanced antimicrobial stewardship and genomic surveillance to mitigate public health risks.

**Supplementary Information:**

The online version contains supplementary material available at 10.1038/s41598-026-59913-w.

## Introduction

*Salmonella enterica* is a globally significant food-borne zoonotic pathogen, with poultry acting as a major reservoir and a key vehicle for transmission to humans through the food chain. Over the last few decades, the emergence of multidrug-resistant (MDR) *S*. *enterica* strains isolated from poultry and poultry-derived products has raised serious concerns in both veterinary and public health sectors. This alarming trend is largely attributed to the widespread and often unregulated use of antimicrobial agents in poultry production, which imposes strong selective pressure favoring the survival and dissemination of resistant strains. The acquisition and spread of antimicrobial resistance (AMR) in *S*. *enterica* are mainly facilitated by a wide array of resistance determinants located on plasmids, integrons, transposons, and other mobile genetic elements, enabling horizontal gene transfer and promoting the dissemination of MDR phenotypes among bacterial populations^1,2^.

In *Salmonella enterica*, the resistance determinants *cat*A1, *cml*A, and *flo*R play key roles in mediating resistance to phenicol antibiotics, including chloramphenicol and florfenicol. The *cat*A1 gene encodes a chloramphenicol acetyltransferase that inactivates chloramphenicol through enzymatic acetylation, whereas *cml*A confers resistance via an efflux pump mechanism that actively exports phenicol compounds from the bacterial cell. In addition, *flo*R encodes a major facilitator superfamily (MFS) transporter that promotes the efflux of both chloramphenicol and florfenicol, thereby reducing their intracellular concentrations. These resistance genes are frequently detected in *S*. *enterica* isolates associated with poultry production, suggesting selective pressure linked to the use of phenicol antibiotics in poultry farming^3^.

The fosfomycin resistance gene *fos*A3 encodes a glutathione S-transferase enzyme that enzymatically modifies and inactivates fosfomycin, leading to reduced susceptibility to this antibiotic. In recent years, *fos*A3 has been increasingly reported in *Salmonella enterica* isolates from poultry, which poses a growing public health concern, particularly as fosfomycin is being reconsidered as an alternative therapeutic option for the treatment of infections caused by multidrug-resistant Enterobacterales^4^. In parallel, tetracycline resistance in *S. enterica* is predominantly associated with efflux pump–encoding genes such as *tet*A and *tet*B, which produce membrane-associated transport proteins that actively export tetracycline molecules out of the bacterial cell, thereby decreasing intracellular drug concentrations and compromising antimicrobial efficacy. These resistance determinants are among the most frequently detected tetracycline resistance genes in poultry-associated *S. enterica* isolates worldwide, especially in regions characterized by intensive poultry production systems^2^.

Quinolone resistance in *Salmonella enterica* is partly mediated by plasmid-associated genes such as *qnr*A and *aac*(6′)-Ib-cr, which typically confer low-level resistance to fluoroquinolones. The *qnr*A gene encodes a pentapeptide repeat protein that protects DNA gyrase and topoisomerase IV from quinolone-mediated inhibition, thereby reducing interference with bacterial DNA replication. In contrast, *aac*(6′)-Ib-cr encodes a variant aminoglycoside acetyltransferase capable of acetylating certain fluoroquinolones, including ciprofloxacin and norfloxacin, resulting in decreased antimicrobial activity^3^. In addition, macrolide resistance in *S*. *enterica* is mainly associated with the erythromycin esterase gene *ere*A, which encodes an enzyme that enzymatically inactivates erythromycin and abolishes its antibacterial effect. Although macrolides are not routinely used for the treatment of salmonellosis in humans, the detection of *ere*A in poultry-associated isolates reflects selective pressure linked to macrolide use in animal production and raises concerns regarding the potential dissemination of resistance determinants among bacterial populations^1^.

Aminoglycoside resistance in *Salmonella enterica* is primarily mediated by genes encoding aminoglycoside-modifying enzymes, including *aac*(3)-IV, *aad*A1, and *aph*A1, which inactivate aminoglycosides through chemical modifications such as acetylation, adenylation, or phosphorylation, reducing the antibiotic’s ability to bind to the bacterial 30 S ribosomal subunit. The plasmid-borne gene *aac*(6′)-Ib-cr, while mainly associated with low-level fluoroquinolone resistance, can also confer resistance to specific aminoglycosides, such as amikacin and kanamycin, via acetylation. Resistance to sulfonamides is mainly mediated by *sul*I and *sul*II, which encode altered forms of dihydropteroate synthase with reduced affinity for the sulfonamides, whereas trimethoprim resistance is conferred by *dfr*A1, producing a resistant variant of dihydrofolate reductase^2,3^.

Class 1 integrons are key mobile genetic elements that facilitate the acquisition and dissemination of antimicrobial resistance in *Salmonella enterica*. They act as genetic platforms capable of capturing and expressing diverse gene cassettes conferring resistance to multiple antimicrobial classes, including aminoglycosides, β-lactams, and trimethoprim–sulfonamide combinations. Although sulfonamide (*sul*I, *sul*II) and trimethoprim (*dfr*A1) resistance genes are often associated with class 1 integrons, cassette composition varies, and not all integrons carry these specific genes. In some isolates, *int*I1 may indicate integrons with alternative cassettes (e.g., *aad*A variants) or truncated forms lacking the typical *sul*I-containing 3′-conserved segment. Moreover, *sul*II is frequently plasmid-borne, and *dfr*A genes may reside on plasmids or transposons independently of integrons, explaining why the presence of class 1 integrons does not always correlate with these resistance determinants^5–8^.

Enterobacterial repetitive intergenic consensus (ERIC)-PCR is a reliable DNA fingerprinting technique widely used to assess the genetic relatedness and diversity of *Salmonella enterica* isolates. By amplifying conserved repetitive intergenic sequences, ERIC-PCR generates strain-specific patterns that differentiate epidemiologically related isolates and trace contamination sources in poultry production systems^9^. When combined with profiling of antimicrobial resistance genes (ARGs), virulence, and biofilm-associated genes, ERIC-PCR provides comprehensive insight into the co-occurrence of resistance and pathogenicity traits. Isolates clustering in ERIC-PCR dendrograms often share similar resistance, virulence, and biofilm gene profiles, suggesting potential epidemiological relatedness and shared genetic characteristics among isolates^9–11^. Integrating ERIC-PCR with molecular analyses of ARGs and virulence factors enhances our understanding of the molecular epidemiology of multidrug-resistant *S*. *enterica*, aiding in risk assessment and evidence-based control strategies in poultry production.

This study aimed to comprehensively characterize the molecular determinants of antimicrobial resistance in *Salmonella enterica* isolated from broiler chicken in Egypt. Specifically, it sought to investigate the genetic relatedness and distribution patterns of resistance genes across different *Salmonella* serotypes, assess serotype-dependent variations in resistance gene profiles, evaluate the association between genotypic resistance determinants and corresponding phenotypic resistance patterns, analyze correlations among resistance genes and their relationship with class 1 integron (*int*I1), and perform molecular typing using ERIC-PCR to explore the genetic diversity of the isolates.

## Results

### PCR detection of antimicrobial resistance genes in *Salmonella* isolates

**Fig. 1:**
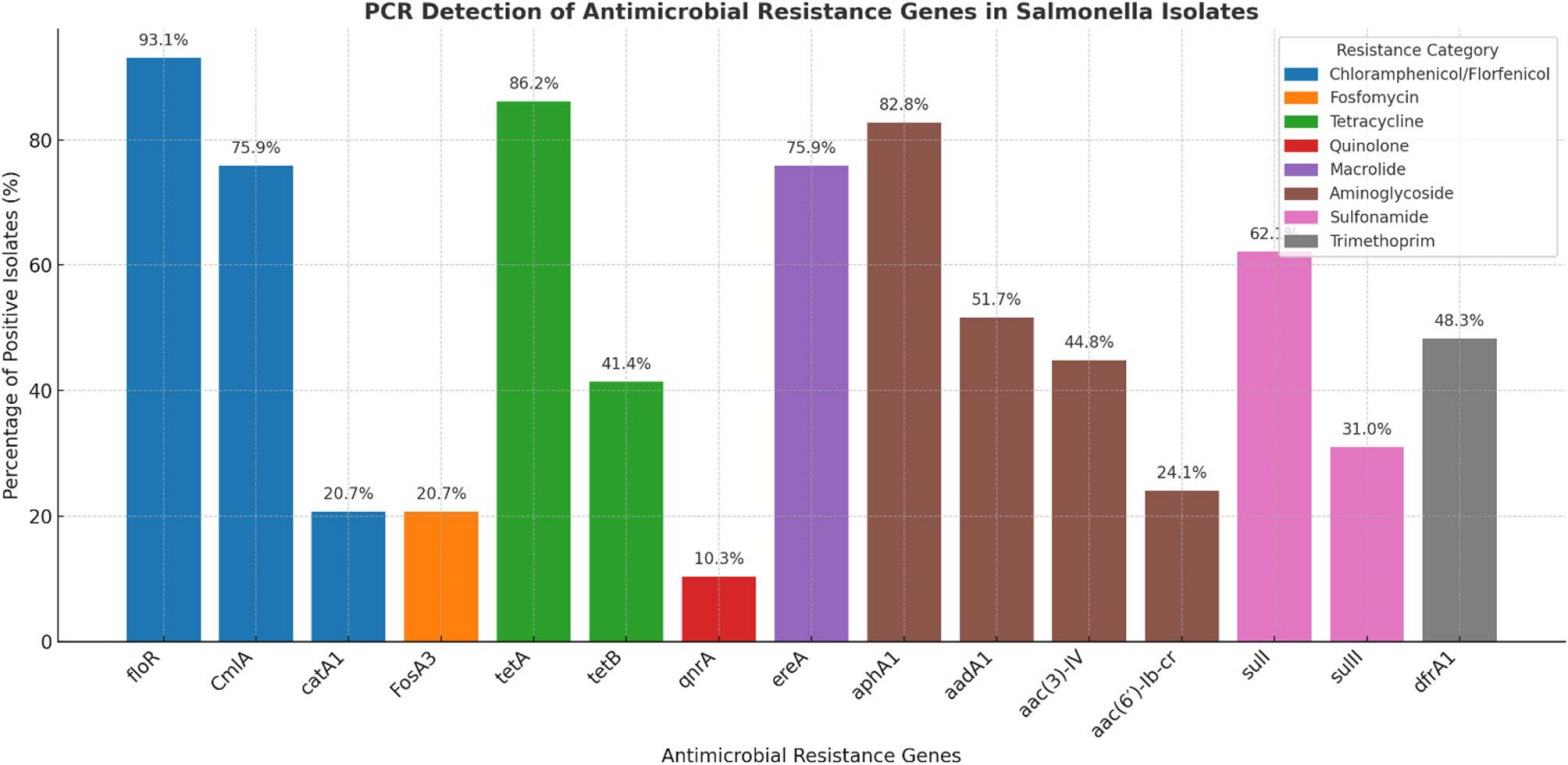
Distribution of antimicrobial resistance genes among *Salmonella* isolates. The figure illustrates the prevalence (%) of each detected resistance gene across all tested isolates.

**Fig. 2:**
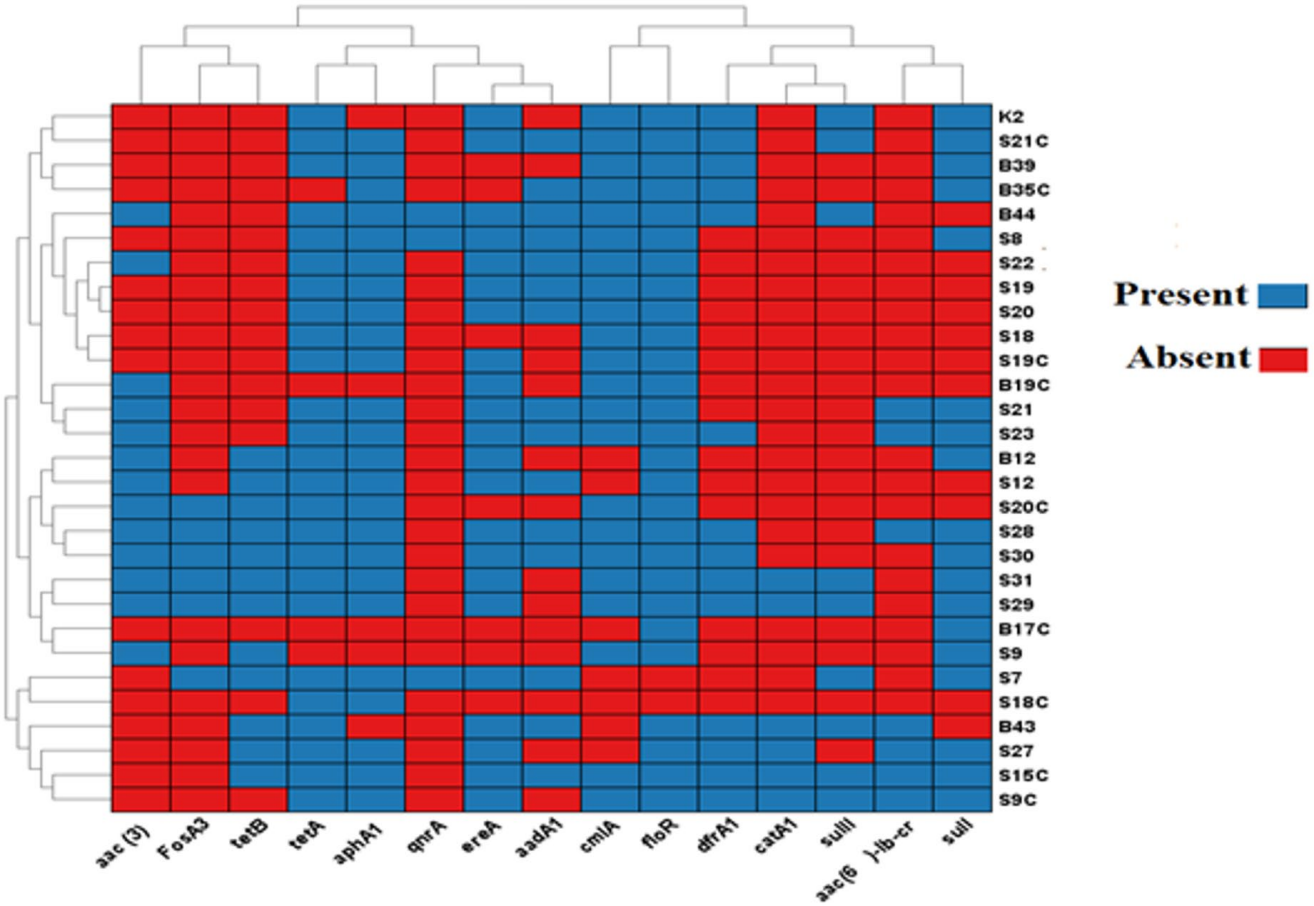
Heatmap showing the distribution of antimicrobial resistance genes across *Salmonella* serovars. Blue indicates gene presence and red indicates gene absence. Both genes and isolates are grouped using hierarchical clustering to illustrate similarity patterns.

The PCR analysis of 29 *Salmonella* isolates revealed the presence of multiple antimicrobial resistance genes (ARGs) distributed among different resistance families. Chloramphenicol/florfenicol resistance genes were highly prevalent, with *flo*R detected in 27/29 (93.1%) isolates, *cml*A in 22/29 (75.9%), and *cat*A1 in 6/29 (20.7%). Fosfomycin resistance genes were represented by *fos*A3, present in 6/29 (20.7%) isolates. Tetracycline resistance genes included *tet*A in 25/29 (86.2%) and *tet*B in 12/29 (41.4%) isolates. Quinolone resistance genes were less frequent, with *qnr*A detected in 3/29 (10.3%) isolates. Macrolide resistance genes were represented by *ere*A, found in 22/29 (75.9%) isolates. Aminoglycoside resistance genes included *aph*A1 in 24/29 (82.8%), *aad*A1 in 15/29 (51.7%), *aac*(3)-IV in 13/29 (44.8%), and *aac*(6′)-Ib-cr in 7/29 (24.1%) isolates. Sulfonamide resistance genes were represented by *sul*I in 18/29 (62.1%) and *sul*II in 9/29 (31.0%) isolates. Finally, the trimethoprim resistance gene *dfr*A1 was detected in 14/29 (48.3%) isolates (Figs. 1 and 2).

### Phylogenetic analysis of antimicrobial resistance genes

**Fig. 3:**
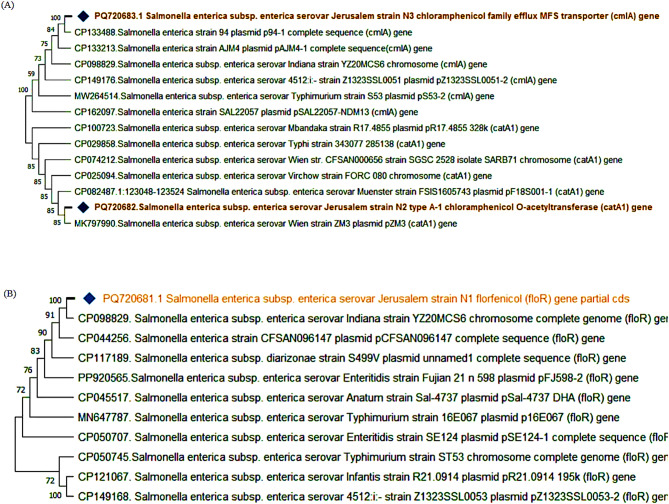
Phylogenetic analysis of chloramphenicol and florfenicol resistance genes in *Salmonella* isolates. **(A)** Phylogenetic relationships of *cml*A and *catA*1 genes showing that the *S*. Jerusalem isolate from this study (marked with a diamond symbol) shares high sequence similarity with corresponding genes from *S*. Indiana, *S*. Typhimurium, and *S*. Virchow. **(B)** Phylogenetic relationships of the *flo*R gene indicating close sequence similarity between the *S*. Jerusalem isolate and sequences from *S*. Indiana, *S*. Enteritidis, and *S*. Typhimurium.

The phylogenetic analyses, based on partial sequences of individual antimicrobial resistance genes, revealed a high degree of sequence similarity and genetic conservation among the determinants identified in the *Salmonella enterica* isolates examined in this study. Chloramphenicol resistance genes (*cml*A and *cat*A1) detected in *S.* Jerusalem clustered with corresponding genes from *S.* Indiana, *S.* Typhimurium, and *S.* Virchow, indicating the widespread distribution of conserved resistance determinants across diverse serovars. Similarly, the *flo*R gene in *S.* Jerusalem exhibited high sequence similarity to homologous genes in *S.* Indiana, *S.* Enteritidis, and *S.* Typhimurium, consistent with the dissemination of these genes via horizontal gene transfer and their association with mobile genetic elements such as plasmids and integrons (Fig. 3).

**Fig. 4:**
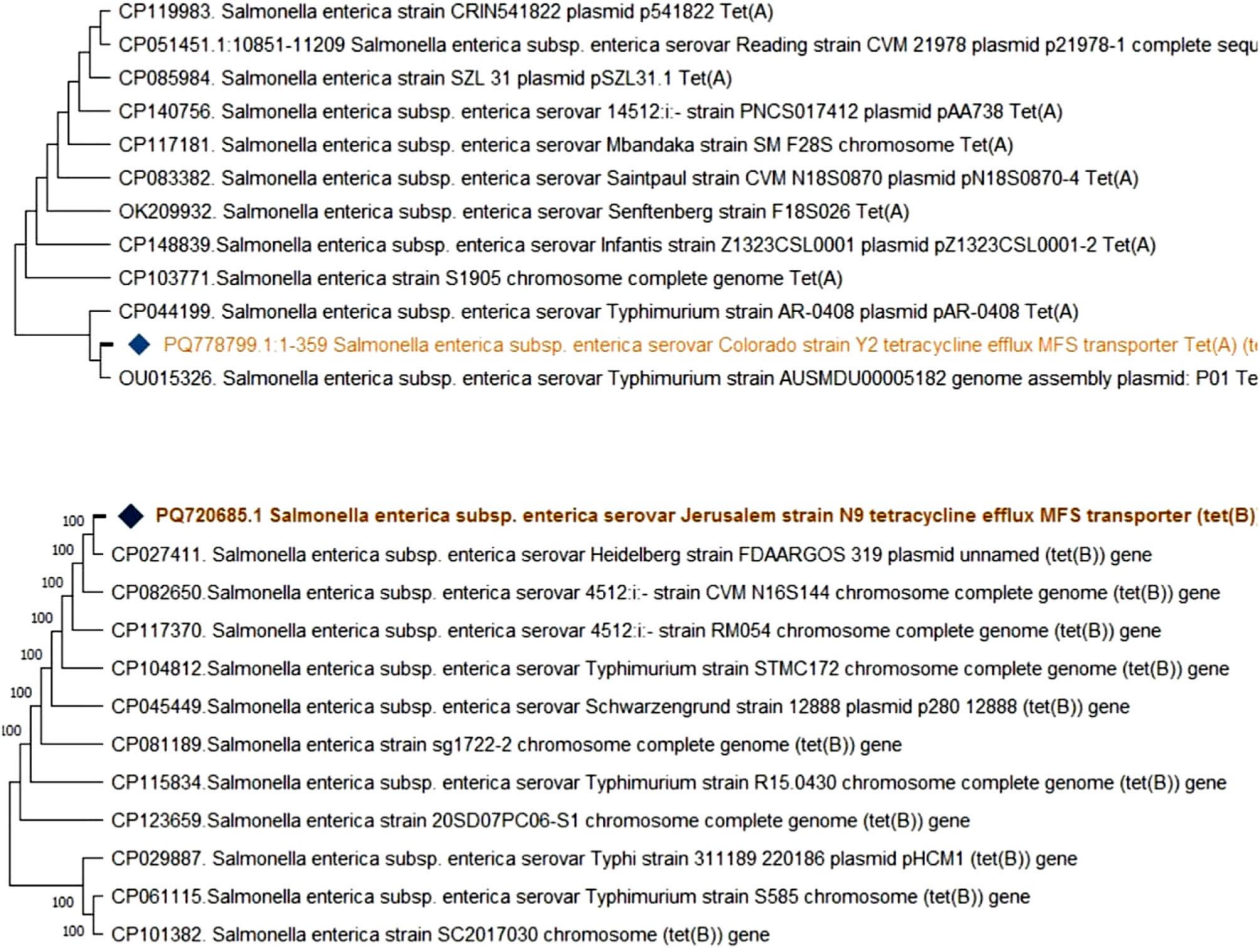
Phylogenetic analysis of tetracycline resistance genes in *Salmonella* isolates. **(A)** Phylogenetic relationships of the *tet*(A) gene showing that the *S*. Colorado isolate from this study (marked with a diamond symbol) shares high sequence similarity with *tet*(A) genes from *Salmonella enterica* strains, particularly serovars Typhimurium and Infantis. **(B)** Phylogenetic relationships of the *tet*(B) gene indicating that the *S*. Jerusalem isolate shows complete sequence identity (100%) with *tet*(B) genes from other *Salmonella* strains included in the analysis.

**Fig. 5:**
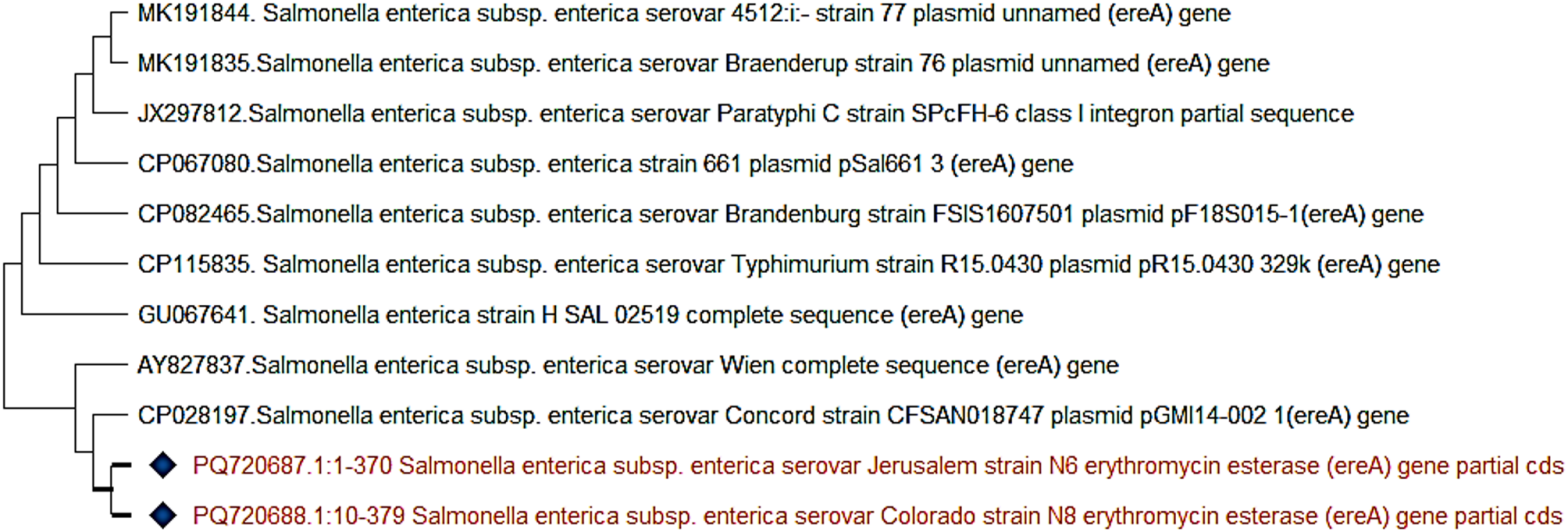
Phylogenetic analysis of the *ere*A gene among *Salmonella enterica* strains. The tree shows that the *S*. Jerusalem and *S*. Colorado isolates from this study (indicated with a diamond symbol) group with strains such as *S*. Typhimurium, *S*. Brandenburg, and *S*. Paratyphi C, indicating high sequence similarity among the analyzed *ere*A gene sequences.

Tetracycline resistance genes demonstrated comparable patterns, where the *tet*(A) gene in *S.* Colorado grouped with sequences from *S.* Typhimurium and *S.* Infantis, while the *tet*(B) gene in *S.* Jerusalem showed 100% identity with other *Salmonella* isolates (Fig. 4). The erythromycin resistance gene *ere*A further supported these observations, with *S.* Jerusalem and *S.* Colorado clustering alongside serovars such as *S.* Brandenburg, *S.* Typhimurium, and *S.* Paratyphi C (Fig. 5).

**Fig. 6:**
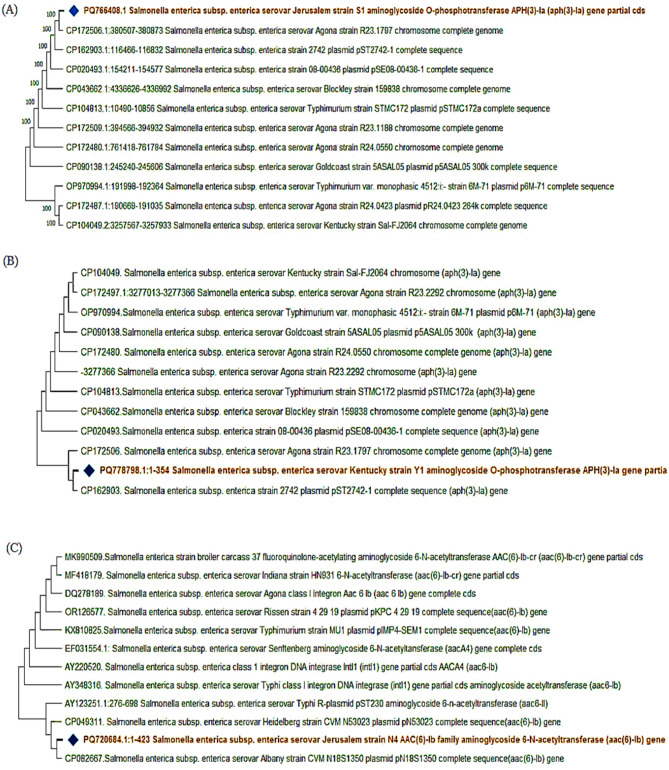
Phylogenetic analysis of aminoglycoside resistance genes in *Salmonella enterica* strains. (**A**) The *aph*(3′)-Ia gene from the *S*. Jerusalem isolate shows complete sequence identity (100%) with corresponding genes from other *Salmonella* strains. (**B**) The *aph*(3′)-Ia gene from the *S*. Kentucky isolate exhibits high sequence similarity with homologous sequences from related *Salmonella* strains. (**C**) The *aac*(6′)-Ib gene from the *S*. Jerusalem isolate groups with other *Salmonella* strains carrying the same gene, indicating high sequence similarity among the analyzed sequences.

**Fig. 7:**
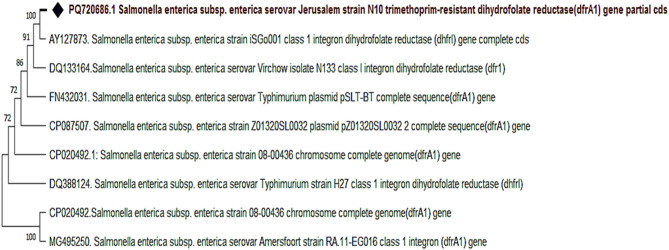
Phylogenetic analysis of the *dfr*A1 gene among *Salmonella enterica* strains. The *S*. Jerusalem isolate from this study groups with other *Salmonella* strains, including *S*. Typhimurium, *S*. Virchow, and *S*. Amersfoort, indicating high sequence similarity among the analyzed *dfr*A1 gene sequences.

Aminoglycoside resistance genes also displayed notable conservation: the *aph*(3’)-Ia gene in *S.* Jerusalem and *S.* Kentucky aligned closely with sequences from other *Salmonella* strains, while the *aac*(6’)-Ib gene in *S.* Jerusalem grouped within a clade of strains carrying the same determinant (Fig. 6). Similarly, analysis of the *dfr*A1 gene showed that *S.* Jerusalem shared high sequence similarity with resistant isolates including *S.* Typhimurium, *S.* Virchow, and *S.* Amersfoort (Fig. 7).

Collectively, these findings indicate that the resistance genes detected in this study are widely distributed and highly conserved across multiple *Salmonella* serovars, highlighting the important role of horizontal gene transfer in shaping the resistome of poultry-associated *Salmonella* populations. However, it should be noted that phylogenetic analysis based on individual resistance genes reflects gene-level relationships and does not necessarily represent the overall evolutionary relationships among bacterial isolates.

### Gene-to-gene correlation of antimicrobial resistance determinants

Spearman’s rank correlation analysis revealed several associations among antimicrobial resistance genes. To control for multiple comparisons, p-values were adjusted using the Benjamini–Hochberg false discovery rate (FDR) method, and only correlations with q < 0.05 were considered statistically significant.

After FDR correction, a limited subset of gene-to-gene correlations remained statistically robust. Notably, *tet*A showed a strong positive correlation with *aph*A1 (*r* = 0.612, q < 0.05), indicating potential co-selection or shared mobile genetic elements. Similarly, *fos*A3 was strongly associated with *tet*B (*r* = 0.608, q < 0.05), suggesting co-occurrence between fosfomycin and tetracycline resistance determinants.

The *cat*A1 gene exhibited multiple significant associations, including strong positive correlations with *sul*II (r = 0.577, q < 0.05) and *dfr*A1 (r = 0.529, q < 0.05), as well as a moderate association with *aac*(6’)-Ib-cr (*r* = 0.508, q < 0.05). These findings suggest potential co-localization of chloramphenicol resistance genes with sulfonamide, trimethoprim, and aminoglycoside resistance determinants.

**Fig. 8:**
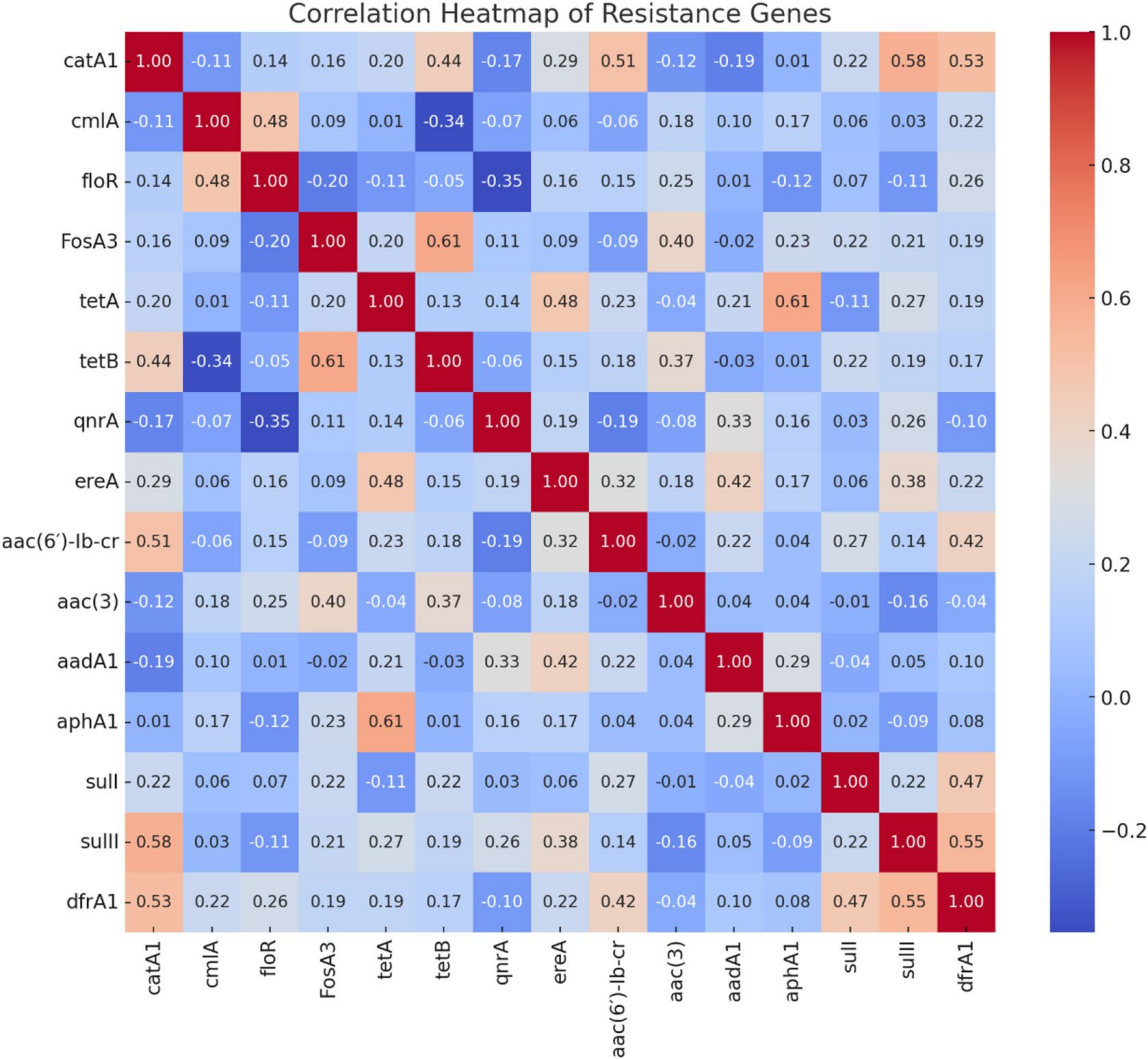
The correlation heatmap shows the relationships among resistance genes, where orange-to-red shades indicate stronger positive associations, while blue shades represent weak or negative correlations.

In addition, *sul*II was positively correlated with *dfr*A1 (*r* = 0.545, q < 0.05), supporting the co-occurrence of sulfonamide and trimethoprim resistance genes. A significant association was also observed between *cml*A and *flo*R (*r* = 0.482, q < 0.05), highlighting a known linkage between chloramphenicol and florfenicol resistance genes (Fig. 8).

In contrast, other correlations observed prior to adjustment, including those involving *ere*A, *aad*A1, *sul*I, and *aac*(3), were no longer statistically significant after FDR correction and should therefore be interpreted with caution (Supplementary Table S1).

### Correlation of resistance genes: current study vs. previous findings

Spearman’s rank correlation analysis revealed multiple associations among the detected antimicrobial resistance genes. To account for multiple comparisons, p-values were adjusted using the Benjamini–Hochberg false discovery rate (FDR) method, and only correlations with q < 0.05 were considered statistically significant.

Notably, *bla*_CTX−M_ exhibited strong positive correlations with *cat*A1 (rs = 0.71, q < 0.01) and *sul*II (rs = 0.67, q < 0.01), indicating consistent co-occurrence among these resistance determinants. In addition, a significant association was observed between *bla*_CTX−M_ and *dfr*A1 (rs = 0.58, q < 0.05).

Similarly, *bla*_CMY−2_ showed a strong positive correlation with *cat*A1 (rs = 0.67, q < 0.01), suggesting potential co-localization of β-lactam and chloramphenicol resistance genes. A significant correlation was also identified between *bla*_OXA−2_ and *qnr*A (rs = 0.56, q < 0.01).

**Fig. 9:**
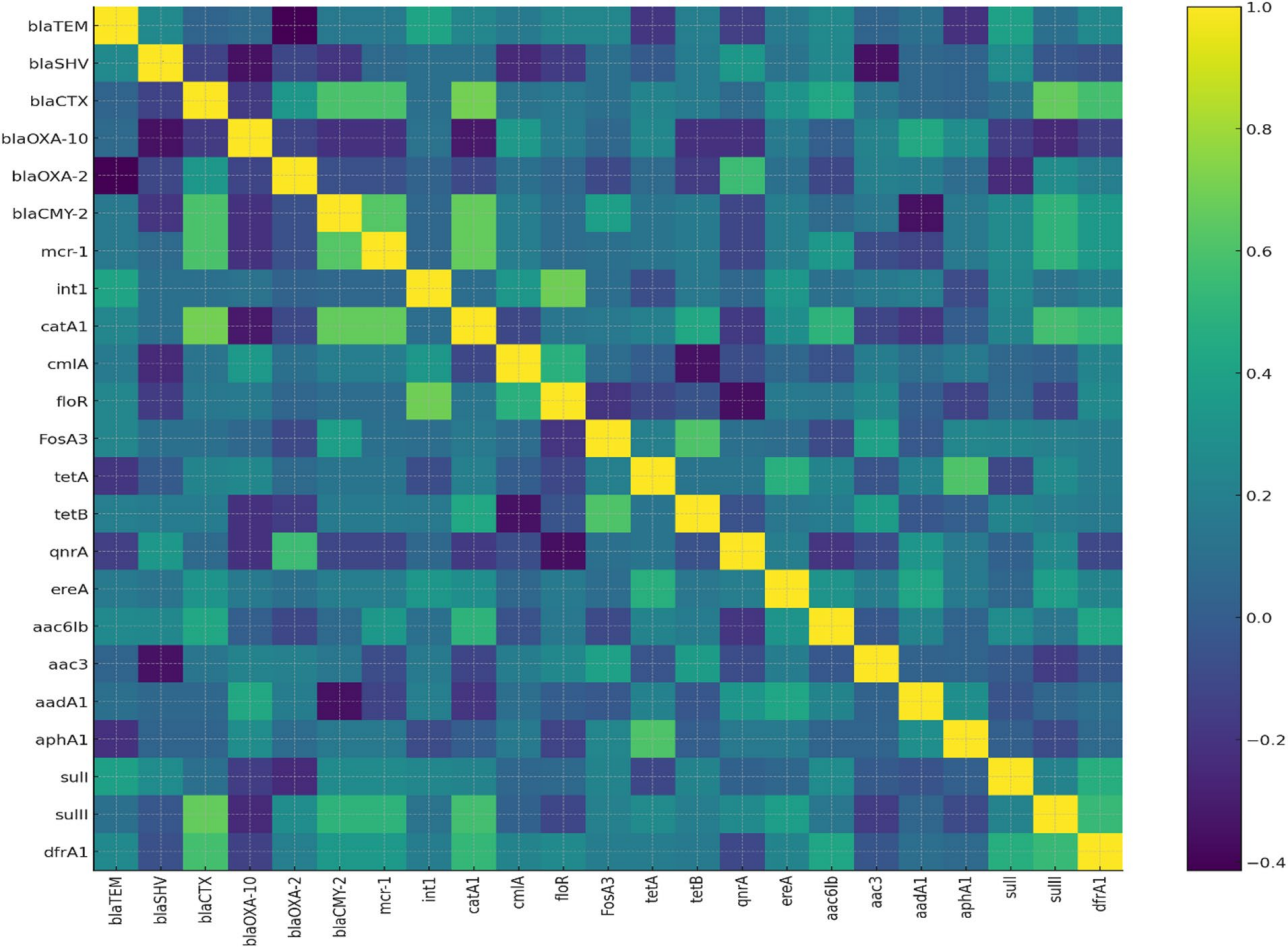
Spearman correlation heatmap shows that gene pairs with stronger correlations appear in green to yellow shades, indicating higher positive association, whereas the darker blue–purple areas represent weak or no correlation between most resistance genes.

Furthermore, the colistin resistance gene *mcr*−1 demonstrated a significant positive association with *cat*A1 (rs = 0.66, q < 0.01), indicating its linkage with multidrug resistance profiles (Table 1) (Fig. 9).

In contrast, other correlations that appeared significant prior to adjustment were no longer statistically significant after FDR correction and should therefore be interpreted with caution (Supplementary Table S2).

**Table 1:** Spearman’s rank correlation coefficients (rs), raw p-values, and FDR-adjusted q-values between β-lactam, colistin, and other antimicrobial resistance genes in *Salmonella* isolates.

Gene 1	Gene 2	Rs	*p*-value	q-value (FDR)
*bla*_CTX−M_	*cat*A1	0.71	< 0.0001	0.002*
*bla*_CTX−M_	*sul*II	0.67	0.0001	0.002*
*bla*_CTX−M_	*dfr*A1	0.58	0.001	0.017*
*bla*_OXA−2_	*qnr*A	0.56	0.00	0.002*
*bla*_CMY−2_	*cat*A1	0.67	0.0001	0.002*
*mcr*−1	*cat*A1	0.66	8 × 10⁻⁵	0.002*

P-values were adjusted for multiple comparisons using the Benjamini–Hochberg false discovery rate (FDR) method. Correlations with q < 0.05 were considered statistically significant.

### Distribution patterns of antimicrobial resistance genes across *Salmonella* serotypes

**Fig. 10:**
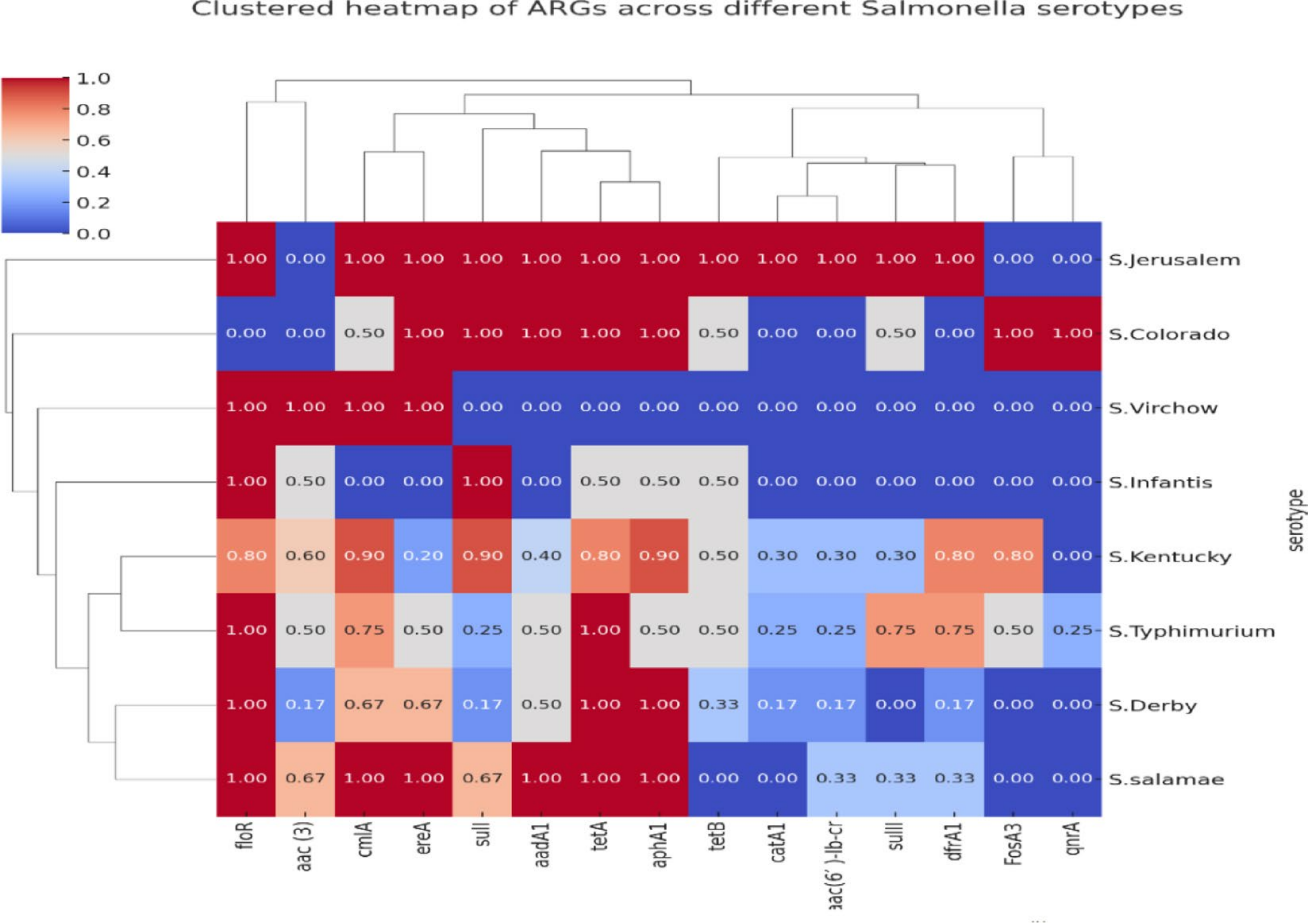
Clustered heatmap illustrating the distribution of antimicrobial resistance genes (ARGs) across different *Salmonella* serotypes. Colors indicate the average presence of each gene within isolates of a given serotype (red = high presence, blue = low presence). Hierarchical clustering reveals co-occurrence patterns of ARGs and highlights serotype-specific gene profiles, suggesting potential associations between certain resistance genes and specific serotypes.

The clustered heatmap generated to visualize the distribution of antimicrobial resistance genes (ARGs) across different *Salmonella enterica* serotypes isolated from broiler samples (Fig. 10) revealed substantial variation in the resistance gene profiles. Overall, *S*. Jerusalem exhibited the highest frequency (1.00) for the majority of the examined ARGs, including *flo*R, *cml*A, *sul*1, *aad*A1, *tet*A, and *aph*A1, indicating a robust multidrug-resistant (MDR) profile. Serotypes *S*. Colorado, *S*. Kentucky, and *S*. Typhimurium also harbored a diverse array of ARGs, albeit with varying frequencies. In contrast, *S*. Infantis, *S*. Virchow, and *S*. Derby displayed a relatively lower frequency and narrower spectrum of resistance determinants, with several genes showing a frequency of 0.00 in *S.* Infantis and generally lower rates in *S.* Derby. The genes *flo*R, *cml*A, *tet*A, and *sul*1 were among the most prevalent across the majority of the serotypes.

### Serotype-dependent distribution of antimicrobial resistance genes in *Salmonella enterica*

The Chi-square test was performed to evaluate the association between *Salmonella* serotypes and the presence of antimicrobial resistance genes. As shown in Supplementary Table S3, most genes exhibited non-significant associations (*p* > 0.05), indicating that their distribution among the examined serotypes was relatively homogeneous. Although a statistically significant association was initially observed between serotype and the *qnr*A gene (χ² = 20.91, df = 7, *p* = 0.0039), this association did not remain significant after applying the Benjamini–Hochberg procedure for multiple testing correction (adjusted *p* = 0.0585). This suggests a potential trend rather than a confirmed association. Notably, *qnr*A, which confers quinolone resistance, was predominantly detected in *S*. Typhimurium and *S*. Colorado, while being absent in most other serotypes.

In addition, the genes *aph*A1, *sul*I, and *dfr*A1 showed p-values close to the significance threshold (*p* = 0.079, 0.052, and 0.059, respectively), but none remained significant after correction, suggesting only tentative associations. Descriptive analysis indicated that these genes were more frequently observed in *S.* Kentucky and *S*. Typhimurium, with occasional detection in *S*. Derby, *S*. Jerusalem, and *S*. Colorado. Specifically, *aph*A1 (aminoglycoside resistance) was commonly identified in *S*. Kentucky and *S*. Typhimurium. Similarly, *sul*I (sulfonamide resistance) and *dfr*A1 (trimethoprim resistance) were more prevalent in the same serotypes, while being less frequent in *S*. Salamae and *S*. Infantis.

**Fig. 11:**
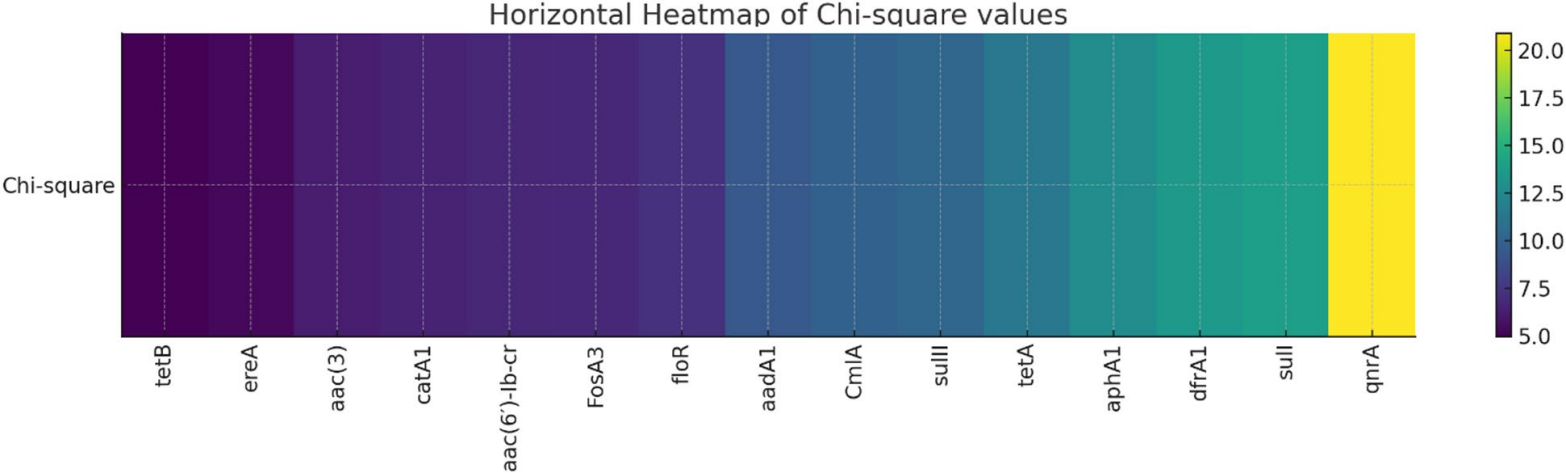
Horizontal heatmap of chi-square values for the association between *Salmonella* serotypes and resistance genes. Higher color intensity reflects stronger associations, with *qnr*A showing the highest chi-square value among the tested genes.

Overall, after correction for multiple comparisons, no statistically significant associations were confirmed between serotypes and resistance genes. However, the observed distribution patterns may indicate possible serotype-related trends that warrant further investigation using larger sample sizes and genomic approaches (Fig. 11).

### Association between genotypic resistance genes and phenotypic resistance patterns

To evaluate whether the *cat*A1 gene contributes to chloramphenicol resistance in the tested isolates, the phenotypic patterns were compared between gene-positive and gene-negative groups. Among the 6 *cat*A1-positive isolates, 5 (83.3%) were resistant, while among the 23 gene-negative isolates, 21 (91.3%) showed phenotypic resistance. Statistical analysis revealed no significant association between *cat*A1 and phenotypic resistance (OR = 0.476; *p* = 0.515). Overall, chloramphenicol resistance was widespread regardless of *cat*A1 carriage. Among the 22 isolates carrying *cml*A, 19 (86.4%) were resistant, while all 7 *cml*A-negative isolates also showed resistance. Statistical analysis indicated no significant association between *cml*A and phenotypic resistance (OR = 0.000; *p* = 0.557), showing that chloramphenicol resistance was widespread regardless of *cml*A carriage. Similarly among the 27 isolates carrying *flo*R, 24 (88.9%) were resistant, while 3 were sensitive or intermediate, and both *flo*R-negative isolates also exhibited phenotypic resistance. The association was not statistically significant (*p* = 1.000), indicating that florfenicol resistance occurred broadly and was not dependent on *flo*R presence (Table 2).

The *fos*A3 gene was detected in 6 isolates, while 23 isolates lacked the gene. All *fos*A3-positive and *fos*A3-negative isolates were phenotypically resistant to fosfomycin; therefore, no statistical association could be evaluated due to the absence of variability in resistance. The *tet*A gene was highly prevalent among the tested isolates, with many resistant strains carrying it. However, some *tet*A-positive isolates were sensitive or intermediate to doxycycline, and statistical analysis showed no significant association (*p* > 0.05). These results indicate that *tet*A acts as a contributing factor rather than a strong predictor of doxycycline resistance. Similarly, the *tet*B gene exhibited comparable resistance patterns between positive and negative isolates, with no significant correlation observed. Overall, although *tet*A and *tet*B are present in several isolates, neither gene appears to play a decisive role in determining doxycycline resistance in this collection of *Salmonella* isolates.

Both *qnr*A and *aac*(6′)-Ib-cr were detected at low frequencies among the isolates; however, their presence did not affect quinolone resistance patterns. Nearly all isolates, regardless of gene status, exhibited high-level resistance to ciprofloxacin and nalidixic acid, preventing meaningful statistical analysis. These results indicate that quinolone resistance in this collection of *Salmonella* isolates is widespread and occurs independently of *qnr*A or *aac(*6′)-Ib-cr. The *ere*A gene was identified in 22 isolates, while 7 isolates lacked it, yet all isolates were resistant to erythromycin, making statistical assessment unnecessary.

The *aac*(6′)-Ib-cr gene had no discernible effect on aminoglycoside resistance among the tested isolates. Although detected in 7 isolates, resistance patterns to streptomycin, amikacin, kanamycin, and apramycin were comparable between gene-positive and gene-negative groups. No statistically significant associations were observed for any of the tested antibiotics (*p* > 0.05), indicating that aminoglycoside resistance occurred independently of *aac*(6′)-Ib-cr. Similarly, the *aac*(3) gene did not show a meaningful impact on aminoglycoside resistance. Despite being present in 13 isolates, resistance patterns to gentamicin, streptomycin, amikacin, kanamycin, and apramycin were largely similar between gene-positive and gene-negative isolates, with all comparisons showing non-significant associations (*p* > 0.05). Overall, these findings indicate that neither *aac*(6′)-Ib-cr nor *aac*(3) serves as a primary determinant of aminoglycoside resistance in this collection of *Salmonella* isolates. Both *aad*A1 (*n* = 15) and *aph*A1 (*n* = 24) genes did not exhibit a significant effect on phenotypic resistance to the tested aminoglycosides (*p* > 0.05). Resistance profiles were comparable between gene-positive and gene-negative isolates, suggesting that the presence of these genes did not confer a discernible phenotypic advantage, likely due to the high baseline prevalence of aminoglycoside resistance within the studied population.

The sulfonamide- and trimethoprim-resistance genes *sul*I (*n* = 18), *sul*II (*n* = 9), and *dfr*A1 (*n* = 14) showed no meaningful association with phenotypic resistance to sulfamethoxazole–trimethoprim (SXT). Resistance was universal across all isolates, including those lacking these genes, precluding statistical comparison. These results indicate that SXT resistance in the examined *Salmonella* population occurs independently of *sul*I, *sul*II, or *dfr*A1.

**Table 2:** Association between genotypic resistance genes and phenotypic resistance patterns.

Gene	No. of isolates with gene (1)	No. of isolates without gene (0)	S + I when gene = 1	*R* when gene = 1	S + I when gene = 0	*R* when gene = 0	%*R* when gene = 1	%*R* when gene = 0	Odds Ratio (OR)	*p*-value	Interpretation
*cat*A1 (C)	6	23	1	5	2	21	83.3%	91.3%	0.476	0.515	No statistically significant association
*cml*A (C)	22	7	3	19	0	7	86.4%	100%	0.000	0.557	No statistically significant association
*flo*R (C)	27	2	3	24	0	2	88.9%	100%	0.000	1.000	No statistically significant association
*fos*A3 (FF)	6	23	0	6	0	23	100%	100%	—	—	Not informative due to no phenotypic variation
*tet*A (DO)	25	4	4	21	1	3	84.0%	75.0%	1.75	0.630	No statistically significant association
*tet*B (DO)	12	17	2	10	3	14	83.3%	82.4%	1.07	0.90	No statistically significant association
*qnr*A (CIP)	3	26	0	3	2	24	100%	92.3%	—	Not computable	Not significant (almost all resistant).
*aac*(6′)-Ib-cr (CIP)	7	22	0	7	2	20	100%	90.9%	—	Not computable	Not significant (phenotypic uniformity).
*qnr*A (NA)	3	26	0	3	0	26	100%	100%	—	Not applicable	No statistical testing possible. all isolates resistant
*aac*(6′)-Ib-cr (NA)	7	22	0	7	0	22	100%	100%	—	Not applicable	No statistical testing possible. All isolates resistant
*ere*A (E)	22	7	0	22	0	7	100%	100%	—	Not applicable	statistical testing was not applicable All isolates were resistant to erythromycin
*aac*(6′)-Ib-cr (S)	7	22	1	6	1	21	85.7%	95.5%	0.29	0.55	No significant correlation with streptomycin.
*aac*(6′)-Ib-cr (AK)	7	22	5	2	17	5	28.6%	22.7%	1.36	0.64	No significant correlation with amikacin.
*aac*(6′)-Ib-cr (K)	7	22	0	7	2	20	100.0%	90.9%	1.83	0.57	No significant association
*aac***(**6′)-Ib-cr (APR)	7	22	2	5	5	17	71.4%	77.3%	0.83	0.72	No significant association
*aac*(3) (CN)	13	16	3	10	3	13	76.9%	81.3%	0.77	0.76	No significant association with gentamicin
*aac*(3) (S)	13	16	1	12	1	15	92.3%	93.8%	0.800	1.000	No significant association
*aac*(3) (AK)	13	16	11	2	11	5	15.4%	31.3%	0.40	0.27	No significant association with amikacin.
*aac*(3) (K)	13	16	2	11	0	16	84.6%	100%	0.14	0.08	No significant association with kanamycin.
*aac*(3) (APR)	13	16	5	8	2	14	61.5%	87.5%	0.23	0.06	No significant association
*aad*A1 (CN)	15	14	2	13	4	10	86.7%	71.4%	2.6	0.16	No significant association with gentamicin resistance.
*aad*A1 (S)	15	14	1	14	1	13	93.3%	92.9%	1.08	1.0	No significant association with streptomycin resistance
*aad*A1 (AK)	15	14	12	3	10	4	20.0%	28.6%	0.63	0.51	No significant association with amikacin resistance.
*aad*A1 (K).	15	14	2	13	0	14	86.7%	100%	0.19	0.09	No significant association with kanamycin resistance.
*aad*A1 (APR)	15	14	5	10	2	12	66.7%	85.7%	0.33	0.10	No significant association (*p* > 0.05).
*aph*A1 (CN)	24	5	6	18	0	5	75.0%	100.0%	0.00	0.553	No significant association. (CN resistance was common in both gene-positive and gene-negative isolates.
*aph*A1 (S)	24	5	2	22	0	5	91.7%	100%	0.82	NS	No significant association (*p* > 0.05). Resistance was high among both gene-positive and gene-negative isolates.
*aph*A1 (AK)	24	5	21	3	3	2	12.5%	40%	0.21	NS	No significant association.
*aph*A1 (APR)	24	5	5	19	2	3	79.2%	60%	0.389	0.616	No significant association. Resistance occurred in both gene-positive and gene-negative isolates.
*sul*I (SXT)	18	11	1	17	0	11	94.4%	100%	0.000	1.000	No significant association; resistance occurred in almost all isolates regardless of sulI presence.
*sul*II (SXT)	9	20	1	8	0	20	88.9%	100%	0.421	0.603	No significant association. Resistance occurred irrespective of *sul*II presence.
*dfr*A1 (SXT)	14	15	1	13	0	15	92.9%	100%	0.000	1.000	No significant association due to high resistance in both gene-positive and gene-negative isolates. Resistance occurred irrespective of *dfr*A1 presence

P-values were not adjusted for multiple comparisons in this table. However, correction using the Benjamini–Hochberg false discovery rate (FDR) method did not identify any statistically significant associations.

### Correlation between integron class 1 (*int*1) and antimicrobial resistance genes

**Fig. 12:**
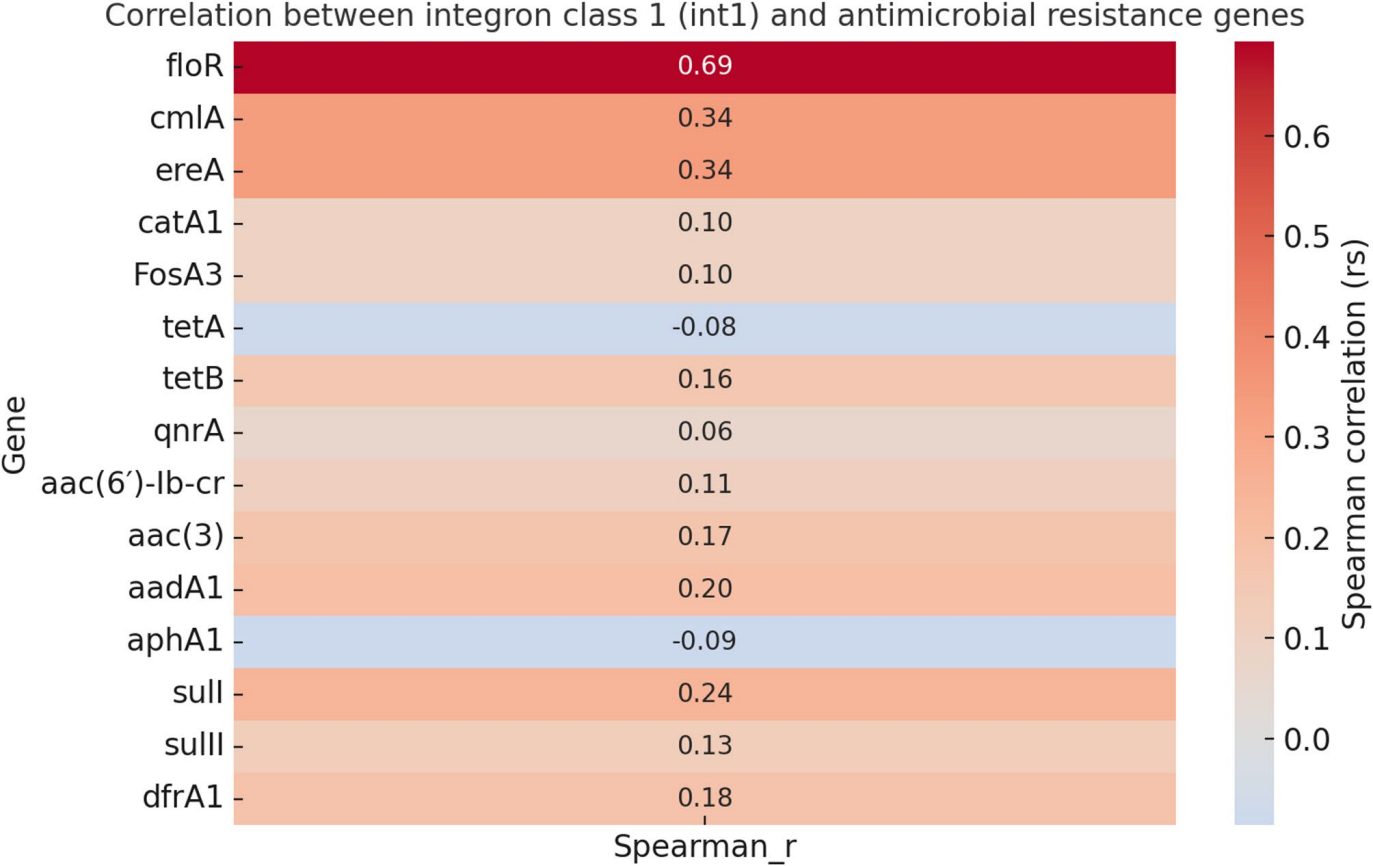
The heatmap shows that the class 1 integron **(***int*I1**)** is most strongly associated with *flo*R, with moderate correlations seen for *cml*A and *ere*A. Other resistance genes show weak or minimal correlation, indicating limited linkage with integron presence.

Spearman’s rank correlation analysis was performed to investigate the association between class 1 integron (*int*I1) and various antimicrobial resistance genes (ARGs) across the bacterial isolates. To account for multiple comparisons, p-values were adjusted using the Benjamini–Hochberg procedure. The analysis revealed a complex pattern of correlations with varying strengths (Fig. 12).

A statistically significant strong positive correlation remained between *int*I1 and the florfenicol resistance gene *flo*R (rs = 0.694, *p* = 3.00 × 10⁻⁵), even after multiple testing correction, indicating that these genetic elements are likely co-located on the same mobile genetic platforms.

Moderate positive correlations were observed between *int*I1 and both *cml*A (rs = 0.335, *p* = 0.0756) and *ere*A (rs = 0.335, *p* = 0.0756); however, these associations did not reach statistical significance either before or after correction, and therefore should be interpreted as indicative trends rather than confirmed relationships.

In contrast, weak and non-significant correlations were observed for the majority of other resistance genes analyzed, including *cat*A1, *fos*A3, *tet*A, *tet*B, *qnr*A, *aac*(6′)-Ib-cr, aac(3), *aad*A1, *aph*A1, *sul*I, *sul*II, and *dfr*A1, all suggesting minimal association with class 1 integron presence.

### ERIC-PCR-based genetic grouping of *Salmonella* isolates

ERIC-PCR genotyping differentiated the 29 *Salmonella* isolates into two major genetic groups (Group A and Group B), comprising seven distinct sub-clusters (A1–A5 and B1–B2). Group A exhibited greater heterogeneity and included five sub-clusters: A1 (S31 – *S*. Kentucky, B12 – *S*. Infantis, and S8 – *S*. Colorado); A2 (S19 – *S*. Derby and K2 – *S*. Typhimurium); A3 (S20 and S18 – *S*. Derby, and S7 – *S*. Colorado); A4 (S18C and S9 – *S*. Kentucky, and S20C – *S*. Typhimurium); and A5 (S19C and S12 – *S*. Derby, B17C – *S*. Infantis, S21C – *S*. Salamae, and S29 – *S*. Kentucky).

**Fig. 13:**
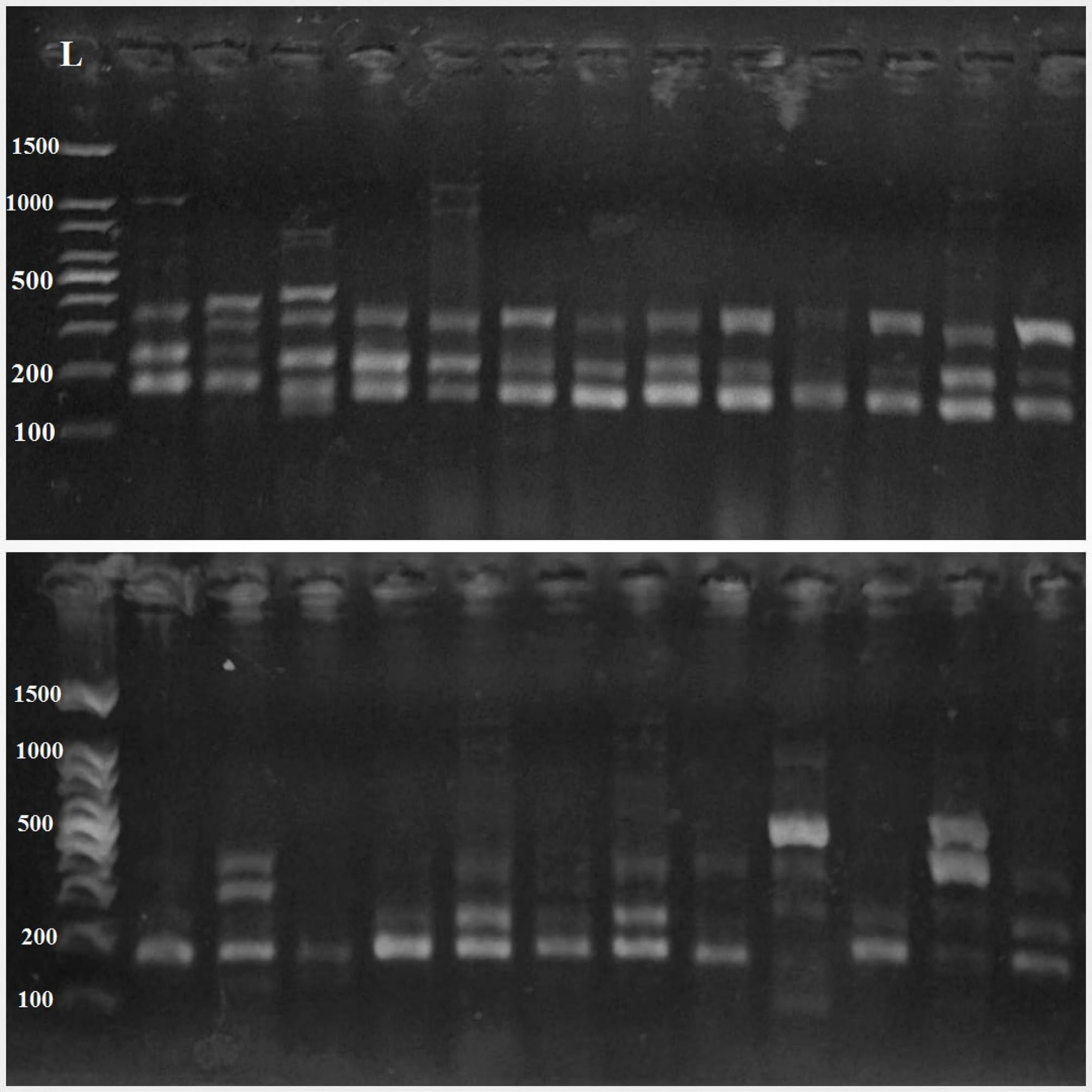
ERIC-PCR banding patterns of *Salmonella enterica* isolates separated on agarose gel. Lane L indicates a 100 bp DNA ladder. The profiles demonstrate variability in band number and distribution among the isolates, reflecting genetic diversity within the examined *Salmonella* population.

**Fig. 14:**
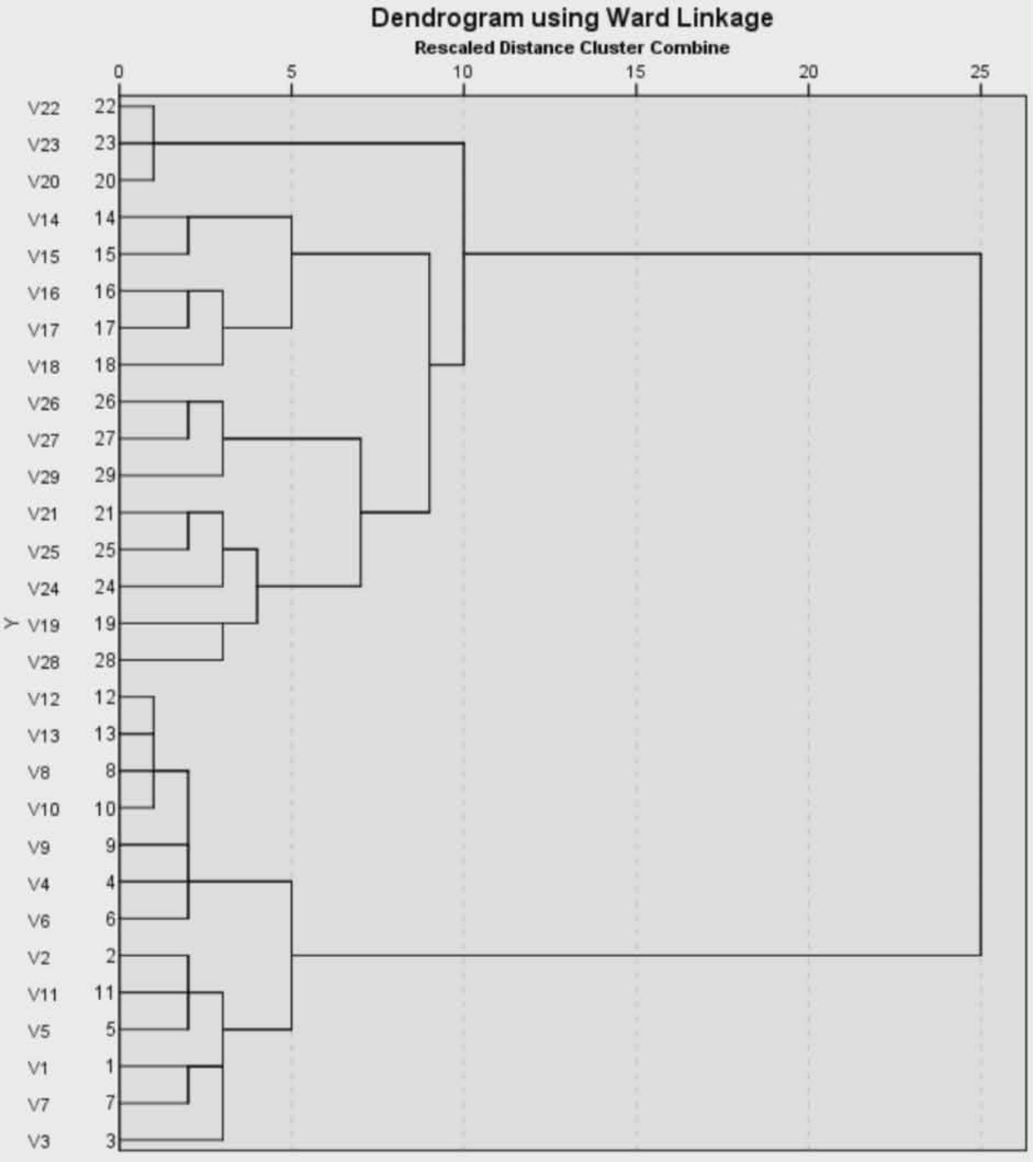
Dendrogram of 29 *Salmonella* isolates generated by ERIC-PCR and Ward linkage, showing two major groups (**A and B**) with seven sub-clusters (A1–A5, B1–B2). Group A is more heterogeneous, while Group B is compact, reflecting partial serotype clustering and genetic diversity among isolates.

Group B comprised two sub-clusters with a more compact clustering pattern: B1 (B44 – *S*. Typhimurium, S22 – *S*. Salamae, S23, S30, S9C, and S28 – *S*. Kentucky, and S27 – *S*. Derby) and B2 (B35C and B39 – *S*. Kentucky, B19C – *S*. Derby, B43 – *S*. Typhimurium, and S15C – *S*. Jerusalem) (Figs. 13 and 14).

**Fig. 15:**
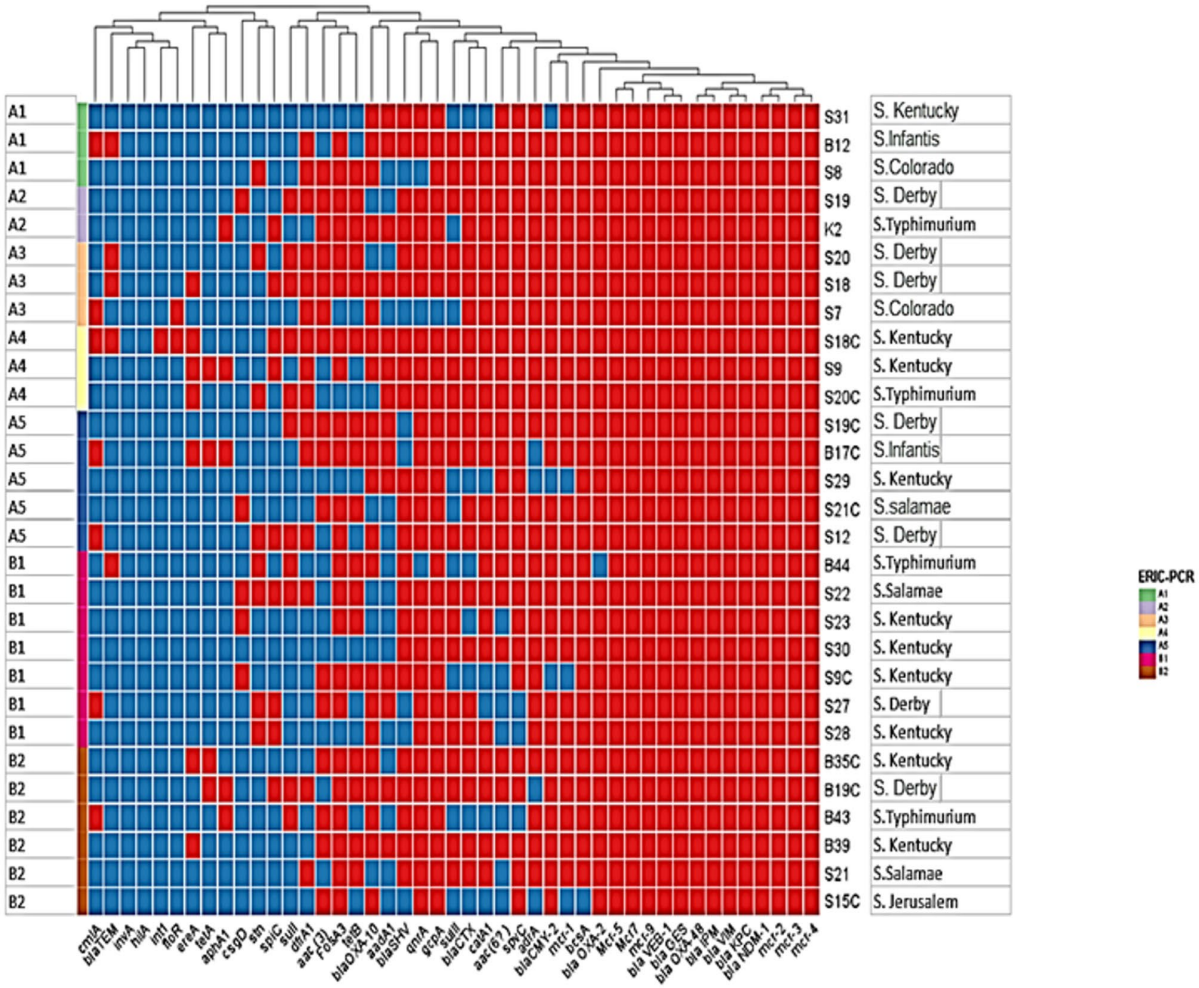
Heatmap showing the presence (blue) and absence (red) of virulence, biofilm, and antimicrobial resistance genes in *Salmonella* isolates, with ERIC-PCR grouping indicated on the left. The dendrogram at the top illustrates the genetic similarity between isolates.

Despite this genetic similarity, substantial variability in antimicrobial resistance (AMR), virulence, and biofilm-associated genes was observed both within and between sub-clusters. This pronounced heterogeneity suggests the contribution of horizontal gene transfer and selective pressure within the poultry production environment, leading to ongoing genetic diversification. Detailed distributions of antimicrobial resistance, virulence, and biofilm genes within each cluster are provided in Fig. 15 and Supplementary Table S4.

## Discussion

In this study, PCR analysis revealed that *Salmonella* isolates carried multiple antimicrobial resistance genes at high frequencies. Resistance determinants for phenicols (*flo*R, *cml*A), tetracyclines (*tet*A), macrolides (*ere*A), aminoglycosides (*aph*A1, *aad*A1, and *aac*-(3)), and sulfonamide/trimethoprim (*sul*I, *dfr*A1) were particularly common, indicating a pronounced multidrug-resistant profile. In our previous study on 29 *Salmonella* isolates, the antimicrobial usage data were collected through structured questionnaires administered to farm owners, workers, and veterinarians at the associated laboratories. These data reflect actual antimicrobial use practices in the field, providing a real-world context for the observed resistance patterns^12^. Florfenicol and thiamphenicol were frequently administered, and this repeated and widespread use is reflected in the high prevalence of florfenicol-associated resistance genes. Similarly, the frequent detection of *tet*A and aminoglycoside resistance genes corresponds with the common use of tetracyclines, apramycin, and gentamicin. The notable occurrence of *ere*A likely results from routine tylosin administration to one-day-old chicks, promoting early-life selection of macrolide-resistant *Salmonella*. *Fos*A3 was detected in 20.7% of isolates, suggesting moderate but emerging selective pressure due to fosfomycin use. In contrast, the low prevalence of *qnr*A (10.3%) aligns with the infrequent or prohibited use of quinolones, such as flumequine and danofloxacin, although occasional off-label use may still occur. The persistent presence of sulfonamide and trimethoprim resistance genes further reflects historic and ongoing usage influencing the resistance landscape. Overall, these findings demonstrate a strong correlation between antimicrobial usage patterns and the emergence and persistence of multidrug-resistant *Salmonella* in poultry farms. This underscores the need for comprehensive stewardship programs, enhanced veterinary oversight, and stricter regulatory measures to curb the dissemination of resistance.

Chloramphenicol (*cml*A, *cat*A1) and florfenicol (*flo*R) are phenicol antibiotics that inhibit bacterial protein synthesis^13^. While chloramphenicol use in food-producing animals is banned in many regions due to severe human toxicities, florfenicol a veterinary analogue is widely used worldwide. Residue and field-use studies also indicate that florfenicol continues to be commonly administered in Egyptian poultry, likely driving the selection of floR-positive strains^14^. In this study, the prevalence of phenicol-resistance determinants was as follows: *cml*A 75.9%, *cat*A1 20.7%, and *flo*R 93.1%. The exceptionally high frequency of floR suggests near-ubiquitous florfenicol resistance, consistent with previous molecular studies in Egypt^15–17^. International studies report more variable *flo*R prevalence, reflecting differences in geographic regions, production practices, and sampling frameworks^13,18^.

The observed disparity between *cml*A (75.9%) and *cat*A1 (20.7%) in our study suggests that multiple phenicol resistance mechanisms coexist in Egyptian *Salmonella*. The widespread presence of *cml*A, which mediates efflux, contrasts with the lower prevalence of *cat*A1, responsible for acetyltransferase-mediated inactivation. This heterogeneity likely reflects differences in the mobile genetic elements (e.g., plasmids, integrons) carrying these genes, as well as variations in local antimicrobial usage patterns in poultry farms, which exert selective pressure favoring certain resistance determinants. Together, these factors contribute to the circulation and predominance of specific resistance genes within Egyptian *Salmonella* populations^19–21^.

From a public-health standpoint, the widespread carriage of *flo*R (93.1%) and high prevalence of *cml*A (75.9%) in broiler *Salmonella* is concerning. Field data collected from the surveyed farms indicate frequent and repeated prophylactic and therapeutic use of florfenicol, which likely maintains strong selective pressure and promotes the persistence and spread of these plasmid-associated genes. Because such plasmids often co-carry additional clinically important resistance determinants (e.g., aminoglycosides, tetracyclines, macrolides, sulfonamides), their dissemination increases the risk of multidrug-resistant zoonotic infections that are difficult to treat in humans. These findings underscore the urgent need for restricting non-therapeutic florfenicol use, implementing targeted molecular surveillance of resistance genes at the farm level, and strengthening reporting systems to monitor and curb the transmission of phenicol resistance to humans.

Fosfomycin, a broad-spectrum antibiotic, inhibits peptidoglycan synthesis via MurA, with resistance mainly mediated by FosA-type enzymes, especially *fos*A3^22^. In this study, *fos*A3 was detected in 20.7% of *Salmonella* isolates from Egyptian broilers, and to the best of our knowledge, this may represent one of the earliest reports of this gene in *Salmonella* from Egyptian poultry. Detection was confirmed using PCR analysis, ensuring accurate identification of the resistance determinant. Field data collected from the surveyed farms indicate that fosfomycin is actively used for therapeutic treatment of *Salmonella* infections, providing a real-world context for the observed resistance. Internationally, high *fos*A3 prevalence has been reported in poultry-associated *E. coli*, including 68% in broilers^23^, 21.9% in ducks^22^, and 4% in Brazilian poultry^24^, reflecting geographic variability influenced by antimicrobial use practices and biosecurity measures. *Salmonella*-focused surveillance generally reports lower *fos*A3 prevalence; for example, 6.5% of 551 isolates in China^25^. The 20.7% prevalence of *fos*A3 observed in this study is higher than previously reported, likely reflecting both the therapeutic use of fosfomycin in the surveyed Egyptian farms and a growing global trend of *fos*A3 dissemination among poultry-associated Enterobacterales. Plasmid-borne *fos*A3, often carried on conjugative Inc-type plasmids, facilitates horizontal transfer to pathogens such as *E. coli* and *Klebsiella pneumoniae*^4,26^, and intensive broiler production conditions further promote rapid dissemination^27^. These findings underscore the urgent need for enhanced genomic surveillance in Egyptian poultry to monitor plasmid spread, co-resistance, and safeguard the clinical efficacy of fosfomycin.

*Tet*A and *tet*B are key tetracycline-resistance genes, primarily mediated by efflux pumps or similar transport mechanisms^2^. In our study, *tet*A was detected in 86.2% of *Salmonella* isolates, while *tet*B was present in 41.4%, indicating a high overall prevalence. This aligns with previous Egyptian studies showing *tet*A dominance: Shabana and Yassin^28^ reported *tet*A in 100% of isolates from retail chicken meat, and Abou Elez et al.^29^ found *tet*A in 83.8% of *Salmonella* from laying hens, table eggs, and humans, whereas *tet*B was detected only in a few isolates. Globally, prevalence varies depending on production systems, sample types, antimicrobial usage, and detection methods. For instance, Brazilian poultry isolates showed *tet*A in 88.66% and *tet*B in 4.12%^30^, while reviews consistently report *tet*A as the most prevalent tetracycline-resistance gene in *Salmonella* spp., followed by *tet*B^31^. The higher *tet*B prevalence observed in our study may reflect local differences in farm management, tetracycline use, sample type, or horizontal gene transfer dynamics. Indeed, field data collected via structured questionnaires from farm owners, workers, and veterinarians indicate frequent tetracycline use in the sampled farms, supporting a selective pressure for both *tet*A and *tet*B. Overall, *tet*A remains the dominant tetracycline-resistance determinant in Egyptian poultry, while *tet*B shows moderate prevalence. These findings highlight the importance of continuous monitoring of both genes to understand resistance dissemination and inform antimicrobial stewardship strategies, minimizing public health risks associated with tetracycline-resistant *Salmonella*.

Plasmid-mediated quinolone resistance (PMQR) genes, including *aac*(6′)-Ib-cr and *qnr*A, reduce fluoroquinolone susceptibility in *Salmonella* and can be horizontally transferred via plasmids within Enterobacterales^3^. In the current study, *aac*(6′)-Ib-cr was detected in 24.1% (7/29) and *qnr*A in 10.3% (3/29) of *Salmonella* isolates, indicating low to moderate circulation of PMQR determinants in the sampled Egyptian poultry farms. These findings are consistent with some previous national reports, where prevalence ranged from 30 to 40% for *aac*(6′)-Ib-cr and 15–20% for *qnr*A in retail meat and broiler farm isolates^32–34^. Internationally, PMQR prevalence varies geographically, with South Korean isolates showing 5.9% and 3.5% for *aac*(6′)-Ib-cr and *qnr*A, respectively^35^, likely due to differences in antimicrobial use patterns, biosecurity measures, and detection methods. The detection of PMQR genes highlights the potential for horizontal gene transfer and underscores the importance of strengthened fluoroquinolone stewardship, stricter regulation, and enhanced genomic surveillance to monitor plasmid-mediated quinolone resistance in Egyptian poultry.

The *ere*A gene, conferring erythromycin resistance, was detected in 75.9% of *Salmonella* isolates in this study. Although erythromycin and tylosin are not used to treat *Salmonella* infections directly, they are commonly administered in Egyptian poultry farms for the prevention and treatment of respiratory diseases, often even on the first day of life to protect chicks from early respiratory challenges. This early and repeated use likely exerts selective pressure on *Salmonella*, contributing to the high prevalence of *ere*A. Local reports on *ere*A in Egyptian poultry are scarce; Shalaby et al.^37^ detected it in only 2 of 19 isolates (10.53%), whereas other studies reported high prevalence in broiler *Salmonella* (~ 90–93%)^38,39^. Our findings suggest this study is among the first to comprehensively evaluate *ere*A in Egyptian poultry *Salmonella*, highlighting the novelty and significance of the data. Globally, *ere*A prevalence in poultry-associated *Salmonella* is low; for example, 4.9% in Bangladesh^40^. Plasmid- and integron-mediated horizontal gene transfer may further contribute to *ere*A gene dissemination within poultry farms.

Aminoglycoside resistance in *Salmonella enterica* appears to be largely driven by aminoglycoside-modifying enzymes (AMEs), as reflected by the detection of *aph*A1, *aad*A1, *aac*(3)-IV, and *aac*(6′)-Ib-cr in our isolates. The predominance of *aph*A1 (82.8%) and *aad*A1 (51.7%) aligns with previous reports linking these genes to integron-mediated resistance in Egyptian poultry, suggesting that integrons continue to play a central role in shaping the aminoglycoside resistance profile^15^. Notably, the detection of *aph*A1, which has not been previously documented in Egyptian poultry-associated *Salmonella*, may indicate recent horizontal gene transfer from co-existing Enterobacterales, particularly under the selective pressure of intensive antimicrobial use. The presence of *aac*(3)-IV and *aac*(6′)-Ib-cr, both commonly plasmid-borne and associated with co-resistance to gentamicin and fluoroquinolones, further underscores the contribution of mobile genetic elements to resistance dissemination. Together, these patterns suggest that aminoglycoside resistance in Egyptian poultry settings is shaped b**y** integron- and plasmid-mediated spread, amplified by co-selection pressures within intensive production environments^41–43^.

In the present study, *sul*I, *sul*II, and *dfr*A1 were detected in 62.1%, 31.0%, and 48.3% of *Salmonella* isolates, respectively, indicating substantial circulation of these determinants in Egyptian poultry. Molecular data from Egypt remain limited, as most studies rely on phenotypic SXT resistance. However, Elkenany et al.^44^ reported *int*I1 in 78.5% of MDR isolates, frequently carrying *dfr*A1 and *sul*I, while Ahmed and Shimamoto^15^ detected class 1 integrons in 42.9% of diseased broilers with multiple *dfr*A variants, although *sul* genes were not examined. These findings highlight the central role of integron-associated gene cassettes in maintaining sulfonamide and trimethoprim resistance locally.

Globally, our data are consistent with reports indicating widespread dissemination of these resistance genes in foodborne *Salmonella*. Thong and Modarressi^45^ detected *sul*I, *sul*II, and *dfr*A1 co-located on class 1 integrons in 63.6% of isolates from retail meats. Forgaciu et al.^46^ documented increasing sulfonamide and SXT resistance over a decade in poultry-derived *Salmonella*, often associated with *sul*I/*sul*II. Similarly, Rafiq et al.^40^ reported *sul*I in 39% of MDR isolates from livestock and poultry products in Bangladesh. Collectively, these patterns suggest that strong selective pressure and integron-mediated horizontal gene transfer drive the high prevalence and co-occurrence of sulfonamide- and trimethoprim-resistance genes in poultry-associated *Salmonella* globally, in agreement with the current findings from Egypt.

In this study, Spearman correlation analysis initially identified several positive associations among antimicrobial resistance (AMR) genes, suggesting non-random co-occurrence and potential co-selection or co-transfer via shared mobile genetic elements. However, after applying the Benjamini–Hochberg false discovery rate (FDR) correction, only a subset of these associations remained statistically robust, emphasizing the importance of controlling for multiple comparisons.

Among the significant correlations, *tet*A exhibited a strong positive association with *aph*A1 (*r* = 0.612, q < 0.05), indicating that tetracycline resistance may contribute to the co-selection of aminoglycoside resistance within the same bacterial population. Such patterns typically arise when these genes are co-located on the same plasmid, transposon, or integron, allowing tetracycline exposure to maintain multidrug-resistance cassettes even in the absence of selective pressure from other antimicrobials. These findings align with previous reports in avian pathogenic *E*. *coli* (APEC) from Egyptian poultry, where *tet*A and aminoglycoside-resistance genes such as *aph*A1 were among the most frequently detected determinants, reflecting widespread multidrug-resistant genotypes^47^.

Similarly, *fos*A3 showed a strong positive correlation with *tet*B (*r* = 0.608, q < 0.05), suggesting co-occurrence between fosfomycin and tetracycline resistance determinants. Plasmid-mediated *fos*A3 has been increasingly reported in Enterobacteriaceae from poultry and waterfowl, frequently alongside multiple resistance genes. For example, 88% of fosfomycin-resistant *E. coli* isolated from waterfowl carried *fos*A3 together with several resistance genes, including tetracycline, aminoglycoside, sulfonamide, quinolone, and β-lactam resistance determinants^48^. Plasmid analyses further show that *fos*A3 is distributed across several incompatibility groups (IncFII, IncI1, IncHI2) and is commonly co-located with ESBL genes such as *bla*_CTX-M_^49^. These findings suggest that selective pressure from commonly used antimicrobials may indirectly maintain fosfomycin resistance by promoting the persistence of multidrug-resistance plasmids^50^. To our knowledge, this is among the first studies to report this association in poultry-derived *Salmonella* from Egypt.

The *cat*A1 gene exhibited multiple significant associations, including strong positive correlations with *sul*II (*r* = 0.577, q < 0.05) and *dfr*A1 (*r* = 0.529, q < 0.05), as well as a moderate association with *aac*(6′)-Ib-cr (*r* = 0.508, q < 0.05). This pattern suggests that chloramphenicol resistance frequently co-occurs with sulfonamide, trimethoprim, and aminoglycoside resistance genes, likely due to their co-localization on shared plasmids or within integron structures. Similar clustering of resistance determinants has been widely reported in multidrug-resistant *Salmonella* from poultry, where multiple ARGs accumulate on mobile genetic elements, enhancing horizontal gene transfer and persistence under antimicrobial selection pressures^51^.

In addition, a strong positive correlation between *sul*II and *dfr*A1 (*r* = 0.545, q < 0.05) supports the well-established co-occurrence of sulfonamide and trimethoprim resistance genes. This is consistent with the genetic organization of class 1 integrons, where these genes are commonly linked and co-transferred via plasmids and transposons in poultry-associated Enterobacteriaceae^52–54^.

The significant co-occurrence of *cml*A and *flo*R (*r* = 0.482, q < 0.05) reflects the well-documented genetic linkage between chloramphenicol and florfenicol resistance determinants. The *flo*R gene, first described on broad-host-range plasmids, is frequently co-located with *cml*A on mobile elements, particularly IncA/C2 and IncHI2 plasmids common in food-producing animals^55,56^. Additionally, *flo*R is often part of multidrug resistance regions such as *Salmonella* genomic island 1 (SGI1), which also harbor *cml*A and other phenicol-resistance genes^57^.

In contrast, other correlations observed prior to FDR correction including those involving *ere*A, *aad*A1, *aac*(3), and *sul*I were no longer statistically significant after adjustment (q > 0.05) and should therefore be interpreted with caution. Although these associations may still reflect potential biological interactions or co-dissemination patterns, further validation using larger datasets and genomic approaches, such as plasmid profiling, whole-genome sequencing, and conjugation assays, is required to confirm these relationships^58^.

In this study, we examined the relationships between resistance genes identified in our previous study on 29 *Salmonella* isolates and other resistance genes detected in the current work. The co-occurrence of β-lactamase and non-β-lactam resistance genes was clearly evident. The strongest associations were observed between *bla*_CTX−M_ and *cat*A1 (r_s = 0.71, q < 0.01), and between *bla*_CTX−M_ and *sul*II (r_s = 0.67, q < 0.01), indicating that ESBL-producing isolates frequently co-harbor chloramphenicol and sulfonamide resistance genes. In addition, a significant association was observed between *bla*_CTX−M_ and *dfr*A1 (r_s = 0.58, q < 0.05), supporting the co-occurrence of trimethoprim resistance within the same genetic background. Such patterns suggest that these determinants may be located on the same plasmid or integron, or on closely linked mobile genetic elements that facilitate their joint transmission. Similar multi-resistance combinations have been documented in *Salmonella* from poultry in Egypt and elsewhere, where ESBL genes commonly coexist with multiple resistance determinants^59^.

For AmpC-type β-lactamase genes, *bla*_CMY−2_ showed a strong positive correlation with *cat*A1 (r_s = 0.67, q < 0.01), suggesting potential co-localization of β-lactam and chloramphenicol resistance genes on shared mobile elements. Previous studies have demonstrated efficient transfer of *bla*_CMY−2_-carrying plasmids among poultry-associated *Salmonella*, emphasizing their role in the dissemination of multidrug resistance through the food chain even in the absence of direct selective pressure^61^.

A significant association was also identified between *bla*_OXA−2_ and *qnr*A (r_s = 0.56, q < 0.01), indicating a possible linkage between β-lactam and quinolone resistance determinants. This co-occurrence may reflect the accumulation of diverse resistance genes on conjugative plasmids, which enhances bacterial adaptability under antimicrobial pressure^60^.

Colistin resistance patterns were also notable. The significant association between *mcr*−1 and *cat*A1 (r_s = 0.66, q < 0.01) indicates that colistin resistance is likely embedded within broader multidrug resistance (MDR) genetic platforms rather than occurring independently. This is consistent with recent reports documenting poultry-associated *Salmonella* carrying *mcr* genes alongside β-lactam, sulfonamide, and other resistance determinants^62^. These findings imply that the use of older antimicrobials such as chloramphenicol may contribute to the maintenance and co-selection of plasmids harboring *mcr*−1, thereby sustaining colistin resistance even in the absence of direct colistin exposure and increasing the potential public health risk through foodborne transmission.

Given that poultry products constitute a major component of the human food supply in Egypt, the detection of *Salmonella* isolates carrying multiple resistance determinants including ESBL, AmpC, colistin, aminoglycoside, sulfonamide, and chloramphenicol genes is highly concerning. These findings indicate a real risk that multidrug-resistant (MDR) strains may reach consumers through the food chain, potentially leading to hard-to-treat infections. The strong gene–gene correlations observed suggest clear patterns of genetic clustering and co-selection driven by antimicrobial use in poultry production, raising serious public health concerns and emphasizing the need for continuous molecular surveillance and prudent antibiotic stewardship.

While these correlations provide compelling evidence of co-occurrence, correlation alone cannot confirm that the resistance determinants reside on the same plasmid, integron, or mobile genetic element. Moreover, PCR detects gene presence but does not reveal expression, gene copy number, or plasmid context. Therefore, further molecular investigations are essential, including plasmid replicon typing, whole-genome or plasmid sequencing to confirm genetic linkage, and screening for integrons and mobile genetic elements. Such advanced analyses will clarify the mechanisms driving co-resistance and support the development of effective intervention strategies to limit the spread of MDR *Salmonella* in the food chain.

The findings of the association between *Salmonella* serovars and antimicrobial resistance genes (ARGs) highlight a critical aspect of the AMR challenge in poultry-associated isolates. In our study, *S*. Jerusalem isolates exhibited an exceptionally rich resistome, carrying a wide spectrum of antimicrobial resistance genes (ARGs) that collectively indicate a highly adapted multidrug-resistant genotype. The detected genes, both in the previous study and in our current work including *bla*_TEM_, *bla*_SHV_, *bla*_CTX−M_, *mcr*−1, *int*I1, *cat*A1, *cml*A, *flo*R, *tet*A, *tet*B, *ere*A, *aac*(6′)-Ib-cr, *aad*A1, *aph*A1, *sul*I, *sul*II, and *dfr*A1cover multiple antibiotic classes and reflect the presence of diverse resistance mechanisms. This extensive ARG repertoire suggests that *S*. Jerusalem has acquired a high capacity for maintaining and disseminating resistance elements, likely supported by mobile genetic elements such as class 1 integrons (*int*I1), which were also detected at a high frequency. Phenotypically, these genotypic traits were strongly mirrored in the antibiotic susceptibility profiles, where *S*. Jerusalem isolate demonstrated resistance to a wide array of antimicrobial agents, including AX, AM, ATM, AMC, FOX, CTR, CAZ, CPM/FEP, CT, C, FF, DO, CIP, NA, E, CN, S, AK, K, APR, and SXT. This broad phenotypic resistance pattern reinforces the functional expression of the detected resistance genes and confirms the emergence of a robust XDR phenotype. Notably, this finding stands in sharp contrast to previously documented *S*. Jerusalem strains from organic poultry flocks in Switzerland and Italy, where antimicrobial susceptibility testing revealed no phenotypic resistance to 13 tested antibiotics despite the serovar being involved in a feedborne outbreak^63^. Such discrepancies underscore the possibility of rapid acquisition and evolution of resistance traits within *S*. Jerusalem, potentially driven by local antimicrobial selection pressure, environmental factors, or introduction of resistance-carrying mobile genetic elements. Together, these observations highlight the emergence of a highly resistant *S*. Jerusalem variant with significant implications for poultry production systems and public health. Ongoing Whole-Genome Sequencing (WGS) will provide deeper insights into the resistome, virulome, and mobilome of this isolate, enabling a better understanding of the genetic basis underlying their expanded MDR profile and guiding future surveillance and control strategies.

The genotypic analysis of *Salmonella enterica* serovar Colorado revealed a highly concerning resistome. Isolate S7 carried *bla*_TEM_, *bla*_SHV_, *int*I1, *fos*A3, *tet*A, *tet*B, *qnr*A, *ere*A, *aad*A1, *aph*A1, *sul*I, and *sul*II, while S8 harbored *bla*_TEM_, *bla*_SHV_, *int*I1, *cml*A, *flo*R, *tet*A, *qnr*A, *ere*A, *aad*A1, *aph*A1, and *sul*I. These genes are often located on mobile genetic elements such as plasmids and class 1 integrons, which facilitate horizontal gene transfer. Phenotypically, these genotypic features were mirrored in the XDR profiles of the isolates, with resistance observed to a broad spectrum of antibiotics including AX, AM, ATM, AMC, FOX, CTR, CAZ, CPM/FEP, CT, C, FF, DO, CIP, NA, E, CN, S, K, APR, and SXT. The high MDRI values (0.9 for S7 and 0.8 for S8) underscore the extensive multidrug resistance of these strains. Notably, *S*. Colorado has rarely been reported in previous studies with such extensive resistance, highlighting the emergence of a potentially high-risk lineage with serious implications for poultry and public health. Whole-genome sequencing is currently underway to fully characterize the resistome and mobilome of these isolates.

Serotypes such as *S*. Kentucky and *S*. Typhimurium are globally recognized for their strong association with MDR and poultry sources. The high frequency of resistance genes in these serovars is particularly alarming, because many of these genes are carried on mobile genetic elements, which facilitate the rapid co-transfer and spread of resistance traits across bacterial populations^64,65^. Conversely, the relatively lower frequency of ARGs in *S*. Infantis, *S*. Virchow, and *S*. Derby observed in our study may reflect localized differences in antibiotic exposure or intrinsic differences in their ability to acquire and maintain mobile genetic elements (MGEs). For instance, *S*. Infantis from poultry has recently been genomically characterized, showing a moderate but notable resistome linked to plasmids, despite not always harboring high ARG loads suggesting selective pressures may vary by region or production system^66^. Additionally, although data are more limited for *S*. Virchow and *S*. Derby, the presence of class 1 integrons and specific resistance determinants in some isolates has been documented, pointing to their latent potential for resistance amplification under the right conditions. Even at lower frequencies, these resistance genes pose an inherent risk: under sustained antimicrobial pressure, they could be mobilized and enriched, facilitating future MDR emergence^67^.

In this study, the analysis of the association between *Salmonella* serotypes and antimicrobial resistance genes indicated that most antimicrobial resistance genes (ARGs) were relatively evenly distributed across the examined serovars, with no statistically significant associations observed after correction for multiple comparisons. Although the *qnr*A gene initially showed a significant association with serotype (χ² = 20.91, *p* = 0.0039), this association did not remain significant after applying the Benjamini–Hochberg false discovery rate (FDR) correction (adjusted *p* = 0.0585). Therefore, this finding should be interpreted as a potential trend rather than a confirmed serotype-specific association.

Descriptively, *qnr*A was more frequently detected in *S*. Typhimurium and *S*. Colorado, while being absent in most other serotypes. As a plasmid-mediated quinolone-resistance determinant, *qnr*A may confer a selective advantage under fluoroquinolone pressure, potentially contributing to its persistence in certain lineages. This observation is partially consistent with previous findings^68^, which reported that plasmid-mediated quinolone resistance (PMQR) genes may cluster within specific *Salmonella* lineages.

Similarly, genes such as *aph*A1, *sul*I, and *dfr*A1, which exhibited p-values close to the significance threshold prior to adjustment (*p* = 0.079, 0.052, and 0.059, respectively), did not remain statistically significant after FDR correction. Nonetheless, descriptive analysis suggested that these genes were more frequently detected in *S*. Kentucky and *S*. Typhimurium, with occasional occurrence in *S*. Derby, *S*. Jerusalem, and *S*. Colorado. These patterns may indicate potential serotype-related tendencies; however, they cannot be considered statistically robust based on the current dataset.

Such tentative trends may reflect the preferential accumulation of resistance genes within certain genetic backgrounds, possibly mediated by class 1 integrons and plasmid-associated elements carrying determinants such as *aph*A1, *sul*I, and *dfr*A1, as previously described^69^.

Overall, the findings suggest that antimicrobial resistance genes are broadly distributed across *Salmonella* serotypes in this study, without strong evidence of serotype-specific clustering. Nevertheless, the observed distribution patterns may highlight emerging tendencies that warrant further investigation using larger sample sizes and higher-resolution genomic approaches, such as whole-genome sequencing, to better understand the potential linkage between serotype and resistance gene dissemination.

The present study provides a comprehensive overview of the distribution of antimicrobial resistance genes (ARGs) and their relationship with phenotypic resistance in *Salmonella enterica* isolates recovered from poultry sources. A key and consistent finding of this work is the pronounced discordance between genotypic determinants and phenotypic expression of antimicrobial resistance across most antimicrobial classes, indicating that resistance in this population cannot be reliably inferred from the detection of individual resistance genes.

Among phenicol resistance determinants, although the *flo*R gene is widely recognized as a major contributor to florfenicol resistance, no statistically significant association was observed between its presence and phenotypic resistance. Interestingly, phenotypic resistance was detected even in *flo*R-negative isolates, while some *flo*R-positive isolates exhibited intermediate or susceptible phenotypes. This suggests that florfenicol resistance in the studied population is not exclusively mediated by *flo*R, but likely results from a multifactorial system involving efflux pump overexpression (e.g., AcrAB–TolC), reduced membrane permeability, and co-selection with other resistance determinants such as *cat*A1 and *cml*A^70–72^. Similar genotype–phenotype inconsistencies involving *flo*R have been reported previously, underscoring that the presence of this gene alone does not reliably predict florfenicol resistance^73,74^.

A similar pattern was observed for chloramphenicol resistance genes *cat*A1 and *cml*A, which showed no statistically significant association with phenotypic resistance. Despite their detection in a subset of isolates, chloramphenicol resistance was nearly ubiquitous regardless of gene carriage. This indicates that the observed resistance phenotype cannot be explained solely by classical enzymatic inactivation genes. Instead, alternative resistance mechanisms, including multidrug efflux systems, additional chloramphenicol/florfenicol resistance determinants, and regulatory mutations, may contribute independently of *cat*A1 or *cml*A^55,71^. Furthermore, resistance genes may be present but not functionally expressed due to weak promoter activity, gene silencing, or genomic context effects^70^.

For fosfomycin resistance, the *fos*A3 gene was detected in a limited proportion of isolates; however, phenotypic resistance was observed in all isolates irrespective of gene carriage. The absence of a significant association suggests that fosfomycin resistance in this population is mediated by alternative mechanisms, including chromosomal mutations affecting uptake systems or other plasmid-borne resistance determinants, consistent with previous reports describing fosfomycin resistance in Enterobacteriaceae as a multifactorial trait^75^.

Similarly, tetracycline resistance genes *tet*A and *tet*B were widely distributed among isolates, yet their presence was not significantly associated with doxycycline resistance. This finding is consistent with earlier studies demonstrating that tetracycline resistance is mediated by multiple overlapping mechanisms, including efflux pumps and ribosomal protection proteins, rather than reliance on a single gene determinant^76,77^.

Quinolone resistance determinants, including *qnr*A and *aac*(6′)-Ib-cr, were detected at low frequencies and showed no significant association with ciprofloxacin or nalidixic acid resistance. Notably, nearly all isolates exhibited high-level quinolone resistance, strongly suggesting that chromosomal mutations within the quinolone resistance–determining regions (QRDRs) of *gyr*A and *par*C represent the primary drivers of resistance, as previously reported in poultry-associated *Salmonella*^78,79^. Further molecular characterization targeting these mutations is warranted and will be addressed in future studies.

Aminoglycoside resistance genes, including *aac*(3), *aad*A1, and *aph*A1, were frequently detected but showed no significant association with phenotypic resistance to gentamicin, streptomycin, amikacin, kanamycin, or apramycin. This suggests that aminoglycoside resistance is not governed by single determinants but rather by combined effects of multiple mechanisms, including additional aminoglycoside-modifying enzymes, efflux systems, and chromosomal mutations^1,80,81^.

Similarly, sulfonamide and trimethoprim resistance genes *sul*I, *sul*II, and *dfr*A1 did not reliably predict phenotypic resistance to sulfamethoxazole–trimethoprim (SXT). Despite their widespread presence, all isolates exhibited phenotypic resistance, suggesting the contribution of alternative resistance alleles, plasmid-associated multidrug resistance regions, or chromosomal mutations, in agreement with previous reports in food-producing animals^82–84^.

Overall, the present study demonstrates a notable discordance between antimicrobial resistance genotypes and phenotypes in *Salmonella*. While a broad array of ARGs was detected, their presence did not consistently correlate with phenotypic resistance at the statistical level. Importantly, this lack of significant association should not be interpreted as evidence that these genes are functionally irrelevant. Rather, it reflects the complexity of resistance expression in populations where phenotypic resistance is already widespread, including among isolates lacking specific ARGs. Similar genotype–phenotype mismatches have been extensively reported and are commonly attributed to variable gene expression, regulatory architecture, plasmid copy number, promoter strength, heteroresistance, and the contribution of alternative mechanisms such as efflux systems and chromosomal mutations^85,86^.

A critical observation in this study is the lack of statistically significant associations in most genotype–phenotype comparisons, which can be largely attributed to the high baseline prevalence of phenotypic resistance among the analyzed *Salmonella* isolates. This limited phenotypic variability results in a ceiling effect that reduces the statistical power of association analyses and restricts the detection of meaningful genotype–phenotype relationships. In addition, although the antimicrobial panel used follows standard surveillance guidelines, the near-universal resistance observed in this multidrug-resistant population suggests that it may not have been sufficiently discriminatory to capture finer differences in resistance expression.

In this context, the main contribution of this study lies in demonstrating that, in a high-MDR *Salmonella* population from poultry in Egypt, phenotypic resistance is largely decoupled from the presence of individual ARGs. This highlights that antimicrobial resistance in such settings is not driven by single-gene effects, but rather by complex and interconnected resistance networks involving multiple genetic, regulatory, and chromosomal factors.

Collectively, these findings highlight the limitations of relying exclusively on PCR-based detection of resistance genes in highly resistant field isolates, where phenotypic and physiological adaptations may play a more prominent role in determining resistance profiles. Future studies integrating whole-genome sequencing, transcriptomic analysis, and investigation of regulatory and chromosomal mutations are essential to fully elucidate the mechanisms underlying antimicrobial resistance in such systems.

Class 1 integrons (*int*I1) are key mobile genetic elements that facilitate the horizontal acquisition and dissemination of antimicrobial resistance genes (ARGs), particularly under the high antimicrobial selection pressure characteristic of poultry production systems^85–87^. In this study, correlation analysis demonstrated a statistically significant strong association between *int*I1 and the florfenicol resistance gene *flo*R (rs = 0.694, q < 0.05). Although *flo*R is not a classical integron cassette, its frequent co-occurrence with *int*I1 is biologically plausible, as *flo*R is widely documented on IncA/C, IncF, and IncI plasmids and transposons that also commonly harbor class 1 integrons^54,74,88–91^. This stable linkage pattern underscores the important role of integrons and associated mobile elements in mediating the spread of florfenicol resistance, a widely used and clinically significant veterinary antimicrobial in poultry production^92–94^.

By contrast, classical integron-associated cassettes such as *aad*A1 and *dfr*A1 exhibited weak and non-significant correlations with *int*I1. This observation does not necessarily indicate the absence of biological linkage but more likely reflects the extensive structural variability of class 1 integrons, differences in cassette array composition, and ecological or selective factors that influence cassette retention^95,96^. Similarly, although *sul*I is typically located within the conserved 3′ segment (3′-CS) of class 1 integrons, it did not display a significant correlation with *int*I1 in the present dataset. Such patterns have been described previously and may result from alternative sulfonamide-resistance mechanisms such as plasmid-borne *sul* genes or from structural modifications including integron truncation, IS26-mediated rearrangements, or deletions of the 3′-CS region^97,98^. These explanations are consistent with the high genetic diversity reported among circulating integrons in Enterobacterales globally^99^. Moderate but non-significant correlations observed for *cml*A and *ere*A suggest possible co-carriage on integron-associated plasmids, as both genes have been reported within the accessory regions of class 1 integrons or on plasmids that commonly co-exist with integrons^54–100^. In contrast, the weak and biologically minimal correlations recorded for tet*A*, *qnr*A, and *aac*(6’)-Ib-cr indicate that these determinants are likely disseminated through mobile genetic elements other than class 1 integrons, most notably diverse plasmid backbones (e.g., IncX, IncHI, IncF) and transposons known to circulate widely in *Salmonella*^58–87^.

From a One Health standpoint, the co-occurrence of *int*I1 with florfenicol, chloramphenicol, and macrolide resistance genes is particularly concerning, as such mobile platforms facilitate gene exchange among commensal and pathogenic bacteria within poultry production and may contribute to transmission to humans through the food chain^101^. However, these findings should be interpreted in light of certain methodological considerations. PCR-based detection cannot resolve the full cassette array structure, distinguish chromosomal from plasmid-borne integrons, or identify specific plasmid incompatibility groups. Therefore, future work integrating whole-genome sequencing and plasmid profiling will be essential to more precisely characterize the genetic context and mobility pathways of ARGs in these isolates.

In this study, ERIC-PCR genotyping successfully differentiated the 29 *Salmonella enterica* isolates into two major genetic groups (Group A and Group B) comprising seven distinct sub-clusters, highlighting substantial genomic diversity within poultry farm environments. This clustering pattern is consistent with previous studies indicating that ERIC-PCR is a reliable and discriminatory molecular tool for assessing genetic relatedness and for epidemiological typing of *Salmonella* in poultry production systems^9–102^.

Despite clear segregation by ERIC-PCR, comparative analysis of antimicrobial resistance (AMR), virulence, and biofilm-associated genes revealed extensive heterogeneity within and across sub-clusters. Such discordance between genetic background and accessory genome content is well documented and reflects the major role of horizontal gene transfer (HGT) in shaping *Salmonella* populations under farm-level selective pressures^103,104^. The marked differences observed among isolates sharing identical ERIC patterns particularly in Patterns A1–A5 underscore the plasticity of the accessory genome and the influence of mobile genetic elements (MGEs), including class 1 integrons, IS elements, and conjugative plasmids.

Isolates in Pattern A1, for example, exhibited identical ERIC-PCR fingerprints yet carried distinct repertoires of ESBL genes, plasmid-mediated quinolone resistance genes, and chloramphenicol/florfenicol resistance determinants. This is consistent with recent studies showing that genetically related *Salmonella* isolates in poultry can acquire different AMR modules through MGE-mediated events, resulting in highly variable resistomes within the same genetic background^65^. Similarly, the detection of *mcr*−1 in selected isolates from both Groups B1 and B2, despite their genetic separation, highlights the widespread circulation of *mcr*-bearing plasmids across the poultry sector, a phenomenon increasingly reported in Africa, Asia, and Europe^105,106^.

In the present analysis, previously generated virulence-gene data from our laboratory were incorporated to better understand the pathogenic potential of the *Salmonella* isolates^107^. Virulence profiling showed that the genes *inv*A, *hil*A, and *spi*C were widely present among the isolates, indicating a strong capacity for host-cell invasion and intracellular survival. Notably, these virulence determinants frequently co-occurred with antimicrobial resistance (AMR) genes, suggesting that the fitness costs typically associated with multidrug resistance are minimal or effectively compensated. This pattern is consistent with previous whole-genome sequencing studies reporting that virulence and resistance determinants are often co-located on the same plasmids or genomic islands, enabling *Salmonella* to retain both pathogenicity and resistance traits simultaneously^108^.

In the present analysis, previously generated biofilm-related data from our laboratory were used to contextualize the genomic and phenotypic characteristics of the *Salmonella* isolates^107^. The detection of major biofilm-associated genes (*csg*D, *adr*A) across multiple lineages highlights the strong biofilm-forming potential of these strains. Biofilm formation represents a critical survival mechanism that enables *Salmonella* to persist in poultry farm environments, colonize equipment and processing surfaces, and facilitate horizontal gene transfer between environmental and pathogenic lineages. The co-occurrence of key biofilm regulators with ESBL and mcr genes in high-risk isolates such as S15C and S29 is particularly concerning, as biofilms provide a protected ecological niche that promotes the maintenance and dissemination of MDR and XDR populations, ultimately posing significant challenges to food safety and public health^109^.

Moreover, while ERIC-PCR offers valuable insights into genetic relatedness, the pronounced heterogeneity within ERIC clusters observed here indicates that *Salmonella* populations in Egyptian poultry production systems undergo continuous genomic remodeling. This diversification is likely driven by antimicrobial pressure, environmental persistence, and frequent exchange of mobile genetic elements. Collectively, these findings underscore the need for an integrated genomic surveillance framework that combines ERIC-PCR, MLST, plasmidome characterization, and whole-genome sequencing (WGS) to effectively track the emergence and spread of MDR and high-risk *Salmonella* clones within poultry farms.

From a One Health perspective, the findings of this study highlight the interconnected nature of antimicrobial resistance across animal, environmental, and human health sectors. Poultry production systems act as important reservoirs of multidrug-resistant *Salmonella*, facilitating the dissemination of resistance determinants along the food chain through contaminated meat products, farm environments, and processing surfaces. This is consistent with previous reports demonstrating the occurrence of ESBL-producing *Salmonella* in poultry meat and their potential transmission to humans, emphasizing the increasing public health burden associated with foodborne antimicrobial-resistant pathogens^110^. In addition, recent evidence has shown that *Salmonella* in poultry can spread through multiple routes, including feed, water, and fecal–oral transmission, contributing to its persistence in farm environments and dissemination along the production chain^111^. The co-occurrence of antimicrobial resistance, virulence, and biofilm-associated traits observed in this study further increases the potential for persistence and transmission of these strains beyond the farm level.

Importantly, the detection of high-risk resistance determinants raises significant public health concerns, as these genes may compromise the effectiveness of critically important antimicrobials used in human medicine. The ability of these strains to persist in biofilms and acquire additional resistance through horizontal gene transfer further enhances their epidemiological significance. Therefore, integrated surveillance strategies that link veterinary, food safety, and human health sectors are essential to monitor and control the spread of resistant *Salmonella* along the farm-to-fork continuum.

## Conclusion

This study provides a comprehensive genomic and phenotypic overview of antimicrobial resistance determinants circulating among *Salmonella enterica* isolates recovered from broiler chicken in Egypt. The high prevalence of multidrug resistance genes particularly *flo*R, *cml*A, *tet*A, *aph*A1, and *sul*I highlights the substantial selective pressure exerted within poultry production systems and underscores the persistence of broad-spectrum resistance mechanisms across multiple serovars. Phylogenetic analyses revealed strong evolutionary conservation among key resistance genes, with multiple ARGs clustering tightly with homologous sequences from diverse *Salmonella* serovars, suggesting frequent horizontal gene transfer and the circulation of highly conserved resistance determinants within poultry-associated populations.

The distribution of ARGs across serotypes demonstrated pronounced serotype-specific patterns, with *S*. Jerusalem, *S*.Colorado, *S*. Kentucky, and *S*. Typhimurium exhibiting the most enriched resistome profiles. Although most genes showed homogeneous distribution, the significant association between *qnr*A and specific serotypes indicates targeted dissemination of quinolone resistance within defined genetic backgrounds. Gene-to-gene correlation analyses further revealed several strong co-occurrence patterns particularly among chloramphenicol, sulfonamide, trimethoprim, tetracycline, and aminoglycoside resistance genes reflecting their frequent co-localization on mobile genetic elements.

Importantly, the strong correlation between class 1 integrons (*int*I1) and *flo*R, alongside near-significant associations with *cml*A and *ere*A, reinforces the role of integrons as major drivers of multidrug resistance dissemination in poultry-associated *Salmonella*. The extensive clustering between β-lactamase genes (*bla*_CTX-M_, *bla*_CMY-2_) and multiple non–β-lactam resistance determinants further emphasizes the complexity and mobility of resistance platforms in this ecosystem. Importantly, ERIC-PCR genotyping supported these findings by revealing two major genetic groups with distinct sub-clusters, demonstrating that isolates with highly similar ERIC-PCR profiles can still exhibit considerable variability in AMR, virulence, and biofilm-associated genes. This divergence within ERIC-defined groups reinforces the view that horizontal gene transfer, rather than vertical transmission alone, is a major contributor to resistome diversity in poultry-associated *Salmonella* populations.

Collectively, these findings illustrate a highly interconnected resistome in poultry-associated *Salmonella*, shaped by phylogenetic conservation, gene-to-gene correlation networks, ERIC-PCR–defined genetic lineages, horizontal gene transfer, serotype-specific dissemination, and integron-mediated mobilization. This study underscores the urgent need for enhanced antimicrobial stewardship, continuous genomic surveillance, and targeted intervention strategies to curb the spread of multidrug-resistant *Salmonella* along the food production chain and mitigate associated public health risks.

## Materials and methods

### Ethical approval

The study protocol was reviewed and approved by the Research Ethics Committee of the Faculty of Veterinary Medicine, Mansoura University, Egypt (Approval Code: MU-ACUC (VM.PhD.23.10.24)). All experimental procedures were conducted in full compliance with the institutional guidelines and relevant national regulations for the care and use of animals.

### Bacterial isolates

The present study included 29 *Salmonella enterica* isolates, accounting for 6.44% of the examined samples. These isolates were selected from a previously conducted study by our research group^12^, which primarily investigated antimicrobial resistance determinants in poultry-derived *Salmonella*. In total, 450 samples were collected between February and April 2024 from White broiler chickens (aged 1–35 days; body weight range approximately 40 g to 2.2 kg) and Sasso chickens (aged 1–50 days; body weight range approximately 40 g to 3.5 kg) obtained from commercial poultry farms and diagnostic laboratories located in Mansoura and nearby rural districts, Egypt. Birds designated for postmortem sampling were humanely euthanized by manual cervical dislocation performed by trained personnel, in accordance with institutional ethical guidelines. No anesthetic agents were administered prior to the procedure.

The sampled population comprised apparently healthy birds (*n* = 100), clinically diseased birds (*n* = 200), and freshly dead birds (*n* = 150). From these groups, *Salmonella* was isolated from 3 samples (3%) of healthy birds, 16 samples (8%) of diseased birds, and 10 samples (6.7%) of freshly dead birds. With respect to sample origin, 9 isolates (31%) were recovered from cloacal swabs, whereas the remaining 20 isolates (69%) were obtained from internal organs, including the liver, spleen, intestines, heart, and unabsorbed yolk sacs. Despite the relatively limited number of isolates, mainly attributed to restricted access to commercial farms operating under strict biosecurity measures and controlled diagnostic laboratory settings, the obtained isolates were considered adequate and representative for subsequent molecular and phenotypic investigations.

Isolation and identification of *Salmonella* were performed in our previously published study in accordance with the ISO 6579:2002 standard procedures. Briefly, samples were initially pre-enriched in buffered peptone water, followed by selective enrichment in Rappaport–Vassiliadis broth. Enriched cultures were then streaked onto Xylose Lysine Deoxycholate (XLD) agar and MacConkey agar plates. Suspected colonies were subjected to conventional biochemical characterization. Molecular confirmation of the isolates was performed by polymerase chain reaction (PCR) amplification of the conserved *inv*A gene, which is specific for *Salmonella enterica*.

Serological identification was conducted using slide agglutination according to the Kauffmann–White classification scheme at the Serology Unit, Animal Health Research Institute (AHRI), Dokki, Egypt, employing commercially available antisera (SISIN, Berlin; Lillidale, UK). Eight different serovars were identified among the confirmed isolates. *S. Kentucky* was the predominant serovar (10 isolates, 34.5%), followed by *S.* Derby (6 isolates, 20.7%) and *S.* Typhimurium (4 isolates, 13.8%). Additionally, *S.* Salamae was detected in 3 isolates (10.3%), while *S. Colorado* and *S. Infantis* were each identified in 2 isolates (6.9%). *S.* Jerusalem and *S.* Virchow were the least frequently detected serovars, represented by a single isolate each (3.4%). This heterogeneous distribution of serovars is consistent with the naturally diverse epidemiology of *Salmonella* in poultry production systems. Importantly, *S.* Colorado and *S.* Jerusalem were documented serologically for the first time in poultry in Egypt^23^. To further confirm molecular specificity, selected *inv*A amplicons from the previous study were purified and subjected to Sanger sequencing at Microsynth Seqlab GmbH (Göttingen, Germany). Sequence analysis demonstrated 100% identity with reference *Salmonella* sequences deposited in the NCBI BLAST database (https://blast.ncbi.nlm.nih.gov/). The corresponding nucleotide sequences were submitted to GenBank under accession numbers PQ720689 (*S. Colorado* M6), PQ720690 (*S. Jerusalem* M8), and PQ720691 (*S. Colorado* M9)^23^.

Based on the findings of the earlier investigation^23^, antimicrobial susceptibility testing of the 29 *Salmonella* isolates revealed complete resistance to cefoxitin (FOX, 30 µg), cefepime (CPM/FEP, 30 µg), ceftazidime (CAZ, 30 µg), nalidixic acid (NA, 30 µg), erythromycin (E, 15 µg), and fosfomycin (FF, 30 µg). Substantial resistance rates were also observed against aztreonam (ATM, 10 µg), amoxicillin–clavulanic acid (AMC, 30 µg), amoxicillin (AX, 25 µg), ampicillin (AM, 10 µg), ciprofloxacin (CIP, 5 µg), streptomycin (S, 10 µg), and kanamycin (K, 30 µg). Resistance levels ranging from moderate to high were recorded for ceftriaxone (CTR, 30 µg), chloramphenicol (C, 30 µg), doxycycline (DO, 30 µg), gentamicin (CN, 10 µg), and apramycin (APR, 15 µg), whereas intermediate resistance was detected for amikacin (AK, 30 µg). In contrast, the isolates exhibited the highest susceptibility to carbapenems, particularly meropenem (MEM, 10 µg) and imipenem (IPM, 10 µg).

Genotypic characterization performed in the previous study^23^ demonstrated that *bla*_TEM_ was the most prevalent β-lactamase gene among the tested isolates, followed by *bla*_OXA-10_, *bla*_SHV_, and *bla*_CTX-M._ Other β-lactamase genes, including *bla*_CMY-2_ and *bla*_OXA-2_, were detected at lower frequencies, while *bla*_VEB-1_ and all screened carbapenemase-encoding genes were not identified. Regarding colistin resistance, only the *mcr*−1 gene was detected. Furthermore, the class 1 integron gene (int*I1*) showed the highest occurrence among the examined isolates.

### Molecular detection of antimicrobial resistance genes

The presence of selected antimicrobial resistance genes, including phenicol resistance genes (*cat*A1, *cml*A, *flo*R), tetracycline resistance genes (*tet*A, *tet*B), quinolone resistance genes (*qnr*A), macrolide resistance genes (*ere*A), aminoglycoside-modifying enzymes (*aac*(6′)-Ib-cr, *aac*(3)-IV, *aad*A1, *aph*A1), sulfonamide resistance genes (*sul*I, *sul*II), and trimethoprim resistance genes (*dfr*A1), was assessed by conventional PCR using gene-specific primers (Table 3). Genomic DNA was extracted from previously isolated *Salmonella* strains using the boiling method^24^ and used as a template for amplification. Each PCR reaction was carried out in a total volume of 25 µL containing 12.5 µL of DreamTaq Green PCR Master Mix (Thermo Scientific, USA), 1 µL of each primer (10 pmol) (Microsynth, Germany), 5.5 µL of nuclease-free water, and 5 µL of DNA template. Amplifications were performed in an Applied Biosystems 2720 thermal cycler (Thermo Fisher Scientific, USA) using the following program: initial denaturation at 94 °C for 5 min; 30 cycles of denaturation at 95 °C for 30 s, 57 °C for 30 s, and extension at 72 °C for 30 s; followed by a final extension at 72 °C for 10 min.

PCR products were resolved on a 1.5% agarose gel in 1× TBE buffer and stained with ethidium bromide. Electrophoresis was carried out at 80 V for 45 min, and DNA bands were visualized under UV light using a Cleaver Scientific transilluminator (UK). A 100 bp molecular weight marker (Qiagen, USA) was used as a size reference. Appropriate negative controls (nuclease-free water) were included in each run to ensure reaction specificity.

To confirm PCR product specificity, representative amplicons of *cat*A1, *cml*A, *floR*, *tet*A, *tet*B, *ere*A, *aac*(6′)-Ib-cr, *aph*A1 and *dfr*A1 were purified using the QIAquick Gel Extraction Kit (Qiagen, Germany) according to the manufacturer’s instructions. Two representative isolates *(Salmonella* serovar Jerusalem strain M8 and *Salmonella* serovar Colorado strain M6) were selected for Sanger sequencing at Microsynth Seqlab GmbH (Göttingen, Germany). Resulting sequences were aligned using BioEdit Sequence Alignment Editor version 7.2.5 (https://bioedit.software.informer.com/) and compared with reference sequences available in the NCBI database using the BLAST (Basic Local Alignment Search Tool) algorithm (https://blast.ncbi.nlm.nih.gov/). Verified sequences were deposited in GenBank under the corresponding accession numbers and strain designations: *cat*A1 (PQ720682, Jerusalem), *cm*lA (PQ720683, Jerusalem), *flo*R (PQ720681, Jerusalem), *tet*A (PQ778799, Colorado), *tet*B (PQ720685, Jerusalem), *ere*A (PQ720687, Jerusalem; PQ720688, Colorado), *aph*A1 (PQ766408, Jerusalem; PQ778798, Kentucky Y1), *aac*(6′)-Ib-cr (PQ720684, Jerusalem), and *dfr*A1 (PQ720686, Jerusalem) **(**Table 4**)**.

Phylogenetic analysis was performed using MEGA 11 (https://www.megasoftware.net). Multiple sequence alignment was carried out using ClustalW integrated within MEGA 11, and phylogenetic trees were constructed using the Maximum Likelihood method with 1,000 bootstrap replicates to assess tree robustness.

**Table 3:** Oligonucleotide primers used in this study.

Target gene	Primer direction and sequence	Amplicon size (bp)	Encoded	References
*cat*A1 Acetyltransferases *cml*A Transporter resistance	F: AGTTGCTCAATGTACCTATAACC R: TTGTAATTCATTAAGCATTCTGCC F: CCGCCACGGTGTTGTTGTTATC R: CACCTTGCCTGCCCATCATTAG	547 698	Phenicol resistance genes (Chloramphenicol)	^112^
*flo*R	F: TATCTCCCTGTCGTTCCAG R: AGAACTCGCCGATCAATG	399	Phenicol resistance genes (chloramphenicol and florfenicol)	^112^
*fos*A3	F: GCGTCAAGCCTGGCATTT R: GCCGTCAGGGTCGAGAAA	282	Fosfomycin resistance genes	^113^
*tet*A *tet*B	F: GGTTCACTCGAACGACGTCA R: CTGTCCGACAAGTTGCATGA F: CCTCAGCTTCTCAACGCGTG R: GCACCTTGCTGATGACTCTT	577 634	Tetracycline resistance genes (Tetracyclines Doxycycline Minocycline)	^114^
*qnr*A	F: ATTTCTCACGCCAGGATTTG R: GATCGGCAAAGGTTAGGTCA	516	Quinolone resistance genes (Nalidixic acid, Ciprofloxacin, Norfloxacin, and Levofloxacin)	^115^
*ere*A	F: GCCGGTGCTCATGAACTTGAG R: CGACTCTATTCGATCAGAGGC	419	Macrolide Resistance Genes (Erythromycin)	^112^
*aac*(6′)-Ib-cr *aac* (3) – IV *aad*A1 *aph*A1	F: TTGCGATGCTCTATGAGTGGCTA R: CTCGAATGCCTGGCGTGTTT F: CTTCAGGATGGCAAGTTGGT R: TCATCTCGTTCTCCGCTCAT F: TATCCAGCTAAGCGCGAACT R: ATTTGCCGACTACCTTGGTC F: ATGGGCTCGCGATAATGTC R: CTCACCGAGGCAGTTCCAT	482 286 447 600	fluoroquinolones (AMEs) gentamicin and tobramycin streptomycin and spectinomycin kanamycin, neomycin, and gentamicin	^116^ ^112^ ^112^ ^117^
*sul*I *sul*II	F: TGGTGACGGTGTTCGGCATTC R: GCGAGGGTTTCCGAGAAGGTG F: CGGCATCGTCAACATAACC R: GTGTGCGGATGAAGTCAG	789 722	Sulfonamide resistance genes	^117^
*dfr*A1	F: GGAGTGCCAAAGGTGAACAGC R: GAGGCGAAGTCTTGGGTAAAAAC	367	Dihydrofolate Reductase (DHFR) Resistance Genes (Trimethoprim)	^118^
ERIC	F: ATG TAA GCT CCT GGG GAT TCAC R: AAG TAA GTG ACT GGG Gtg AGC G	Variable	Enterobacterial Repetitive Intergenic Consensus	^119^

**Table 4:** Sequence identification and corresponding GenBank accession numbers.

Accession number	Identification
PQ720682	*Salmonella enterica* subsp. enterica serovar Jerusalem strain type A-1 chloramphenicol O-acetyltransferase (*cat*A1) gene, partial cds
PQ720683	*Salmonella enterica* subsp. enterica serovar Jerusalem chloramphenicol family efflux MFS transporter (*cml*A) gene, partial cds
PQ720681	*Salmonella enterica* subsp. enterica serovar Jerusalem florfenicol (*flo*R) gene, partial cds
PQ778799	*Salmonella enterica* subsp. enterica serovar Colorado tetracycline efflux MFS transporter *tet*(A) gene, partial cds
PQ720685	*Salmonella enterica* subsp. enterica serovar Jerusalem tetracycline efflux MFS transporter *tet*(B) gene, partial cds
PQ720687	*Salmonella enterica* subsp. enterica serovar Jerusalem erythromycin esterase (*ere*A) gene, partial cds
PQ720688	*Salmonella enterica* subsp. enterica serovar Colorado erythromycin esterase (*ere*A) gene, partial cds
PQ766408	*Salmonella enterica* subsp. enterica serovar Jerusalem aminoglycoside O-phosphotransferase *aph*(3’)-Ia gene, partial cds
PQ778798	*Salmonella enterica* subsp. enterica serovar Kentucky strain Y1 aminoglycoside O-phosphotransferase *aph*(3’)-Ia gene, partial cds
PQ720684	*Salmonella enterica* subsp. enterica serovar Jerusalem aminoglycoside 6’-N-acetyltransferase *aac*(6’)-Ib gene, partial cds
PQ720686	*Salmonella enterica* subsp. enterica serovar Jerusalem trimethoprim-resistant dihydrofolate reductase *dfr*A1 gene, partial cds

### Molecular typing by ERIC-PCR method

Molecular characterization of 29 *Salmonella* isolates was conducted using ERIC-PCR to evaluate their genetic diversity. Genomic DNA previously extracted from the isolates served as the amplification template. PCR reactions were assembled in a final volume of 25 µL, comprising 12.5 µL of DreamTaq Green PCR Master Mix (Thermo Scientific, USA), 1 µL of each primer (10 pmol) (ERIC-F: 5’-ATG TAA GCT CCT GGG GAT TCA C-3’ and ERIC-R: 5’-AAG TAA GTG ACT GGG GTG AGC G-3’; metabion, Germany), 5.5 µL nuclease-free water, and 5 µL of template DNA. Amplification was carried out in an Applied Biosystems 2720 thermal cycler (Thermo Fisher Scientific, USA) using the following cycling conditions: initial denaturation at 94 °C for 5 min; 40 cycles consisting of denaturation at 91 °C for 1 min, annealing at 25 °C for 2 min, and extension at 72 °C for 2 min; followed by a final extension at 72 °C for 5 min.

PCR products were electrophoresed on 2% agarose gels (Sigma-Aldrich) prepared in 1× TBE buffer at 70 V for 1 h. Bands were visualized under UV illumination using a Cleaver Scientific transilluminator (UK), with a 100 bp DNA ladder (Qiagen, USA) serving as a size reference. Negative controls containing nuclease-free water were included to verify the specificity of amplification. ERIC-PCR profiles were scored based on the presence or absence of DNA bands, and the resulting binary matrix was used for further analysis. Hierarchical clustering was performed using Ward’s method and the unweighted pair group method with arithmetic mean (UPGMA) to construct a dendrogram representing genetic relationships among the isolates. Cluster analysis and dendrogram generation were performed using IBM SPSS Statistics version 22 (IBM Corp., Armonk, NY, USA; https://www.ibm.com/products/spss-statistics). Additionally, pairwise similarity indices, including the Jaccard/Tanimoto coefficient and the number of shared bands between samples, were calculated using the online PlanetCalc Jaccard Similarity Calculator (https://planetcalc.com/1664/).

### Statistical analysis

All statistical analyses were performed using GraphPad Prism version 9.5.1 (GraphPad Software, San Diego, CA, USA; https://www.graphpad.com/) and the Social Science Statistics online platform (https://www.socscistatistics.com/). Categorical variables, including the presence or absence of antimicrobial resistance genes and phenotypic susceptibility patterns, were summarized as frequencies and percentages. The association between *Salmonella* serotypes and antimicrobial resistance genes was evaluated using the Chi-square test of independence; Fisher’s exact test was applied whenever expected cell counts were below five.

The relationship between genotypic resistance determinants and phenotypic resistance (resistant vs. susceptible/intermediate) was assessed by calculating odds ratios (ORs) with 95% confidence intervals, accompanied by Chi-square or Fisher’s exact test depending on data structure. Gene-to-gene associations were examined using Spearman’s rank correlation coefficient. Correlation matrices were generated using Python version 3.10 (https://www.python.org/) with the SciPy (https://scipy.org/) and pandas (https://pandas.pydata.org/) libraries and visualized as heatmaps using **seaborn** (https://seaborn.pydata.org/), while additional heatmaps were prepared using TBtoos version 1.098 (https://github.com/CJ-Chen/TBtools). All statistical tests were two-tailed, and a p-value < 0.05 was considered statistically significant. To control for multiple comparisons, p-values obtained from Spearman correlation and chi-square analyses were adjusted using the Benjamini–Hochberg false discovery rate (FDR) method. Adjusted p-values (q-values) < 0.05 were considered statistically significant.

## Supplementary Information

Below is the link to the electronic supplementary material.

Supplementary Material 1

Supplementary Material 2

## Data Availability

All relevant data are included within the manuscript, and site-specific information is provided in the supplementary material. Additional data may be made available upon reasonable request and with the approval of the corresponding author.The nucleotide sequences generated in this study have been deposited in the NCBI GenBank database under accession numbers PQ720681 (floR), PQ720682 (catA1), PQ720683 (cmlA), PQ720684 (aac(6′)-Ib-cr), PQ720685 (tetB), PQ720686 (dfrA1), PQ720687 and PQ720688 (ereA), PQ766408 and PQ778798 (aphA1), and PQ778799 (tetA). All sequence records are publicly available through the NCBI GenBank database (https://www.ncbi.nlm.nih.gov/genbank/): https://www.ncbi.nlm.nih.gov/nuccore/PQ720681https://www.ncbi.nlm.nih.gov/nuccore/PQ720682https://www.ncbi.nlm.nih.gov/nuccore/PQ720683https://www.ncbi.nlm.nih.gov/nuccore/PQ720684https://www.ncbi.nlm.nih.gov/nuccore/PQ720685https://www.ncbi.nlm.nih.gov/nuccore/PQ720686https://www.ncbi.nlm.nih.gov/nuccore/PQ720687https://www.ncbi.nlm.nih.gov/nuccore/PQ720688https://www.ncbi.nlm.nih.gov/nuccore/PQ766408https://www.ncbi.nlm.nih.gov/nuccore/PQ778798https://www.ncbi.nlm.nih.gov/nuccore/PQ778799.
