# Supplementary material for: Genetic diversity and mobile genetic element associated multidrug resistance in Salmonella enterica from broiler chickens in Egypt
Source: Sci Rep. 2026 Jul 3;16:20548. doi: 10.1038/s41598-026-59913-w (PMC13332045; doi:10.1038/s41598-026-59913-w)
Supplement: Supplementary file 1 — Supplementary Material 1 [file 41598_2026_59913_MOESM1_ESM.docx]

**Table S1.** Spearman correlation coefficients between antimicrobial resistance genes

| Gene 1 | Gene 2 | rs | p-value | q-value (FDR) |
| --- | --- | --- | --- | --- |
| *tet*A | *aph*A1 | 0.612 | <0.001 | 0.013* |
| *fos*A3 | *tet*B | 0.608 | <0.001 | 0.013* |
| *fos*A3 | *aac*(3) | 0.40 | 0.034 | 0.130 |
| *cat*A1 | *sul*II | 0.577 | 0.001 | 0.013* |
| *sul*II | *dfr*A1 | 0.545 | 0.002 | 0.020* |
| *cat*A1 | *dfr*A1 | 0.529 | 0.003 | 0.026* |
| *cat*A1 | *tet*B | 0.44 | 0.018 | 0.078 |
| *cat*A1 | *aac*(6’) | 0.508 | 0.005 | 0.035* |
| *cml*A | *flo*R | 0.482 | 0.008 | 0.048* |
| *tet*A | *ere*A | 0.475 | 0.009 | 0.050 |
| *sul*I | *dfr*A1 | 0.471 | 0.010 | 0.052 |
| *ere*A | *aad*A1 | 0.42 | 0.022 | 0.095 |
| *aac*(6’) | *dfr*A1 | 0.42 | 0.022 | 0.095 |

P-values were adjusted using the Benjamini–Hochberg false discovery rate (FDR) method. Correlations with q < 0.05 were considered statistically significant.

**Table S2.** Spearman’s rank correlation (rs) and p-values between β-lactam, colistin, and other antimicrobial resistance genes in *Salmonella* isolates

| Genes | *cat*A1 | *cml*A | *flo*R | *fos*A3 | *tet*A | *tet*B | *qnr*A | *ere*A | *aac*(6′ )-Ib-cr | *aac* (3) | *aad*A1 | *aph*A1 | *sul*I | *sul*II | dfrA1 |
| --- | --- | --- | --- | --- | --- | --- | --- | --- | --- | --- | --- | --- | --- | --- | --- |
| *bla_TEM_* | rs= 0.23  p= 0.22 | rs= 0.17 p = 0.38 | rs= 0.24  p= 0.22 | rs= 0.23  p =0.22 | rs=  -0.18  p= 0.34 | rs= 0.2  p= 0.30 | rs=-0.14  p= 0.45 | rs= 0.17 p= 0.38 | rs= 0.26  p= 0.18 | rs= 0.04  p= 0.82 | rs= 0.11 p= 0.58 | rs=  -0.21  p= 0.28 | rs = 0.4 p= 0.03 | rs = 0.11  p= 0.57 | rs= 0.26  p= 0.18 |
| *bla*_SHV_ | rs= 0.11  p= 0.57 | rs= -0.25 p= 0.2 | rs= -0.16  p= 0.39 | rs= 0.11 p= 0.57 | rs = -0.01 p= 0.97. | rs= 0.18  p= 0.35 | rs= 0.34 p= 0.07 | rs= 0.13 p= 0.50. | rs= 0.25 p= 0.2. | rs = -0.35 p= 0.07. | rs= 0.06 p= 0.75. | rs= 0.04 p= 0.82 | rs= 0.27 p= 0.15. | rs = -0.03 p= 0.88 | rs = -0.06 p= 0.75 |
| *bla*_CTX-M_ | rs=  0.71  p=  <0.0001 | rs= 0.13 p=  0.50 | rs=  0.15 p= 0.43 | rs=  0.11 p= 0.57 | rs=  0.23 p= 0.24 | rs=  0.18 p= 0.35 | rs=  0.07 p= 0.70 | rs=  0.31 p= 0.09 | rs=  0.42 p= 0.02 | rs=  0.14 p= 0.47 | rs=  0.06 p= 0.75 | rs=  0.04 p= 0.82 | rs=  0.11 p= 0.57 | rs=  0.67 p= 0.0001 | rs=  0.58 p= 0.001 |
| *bla*_OXA-10_ | rs =  -0.32 p= 0.1 | rs= 0.35 p= 0.06 | rs= 0.17 p= 0.38 | rs= 0.066  p= 0.74 | rs= 0.25  p= 0.196 | rs=  -0.21  p= 0.29 | rs= -0.21 p= 0.28 | rs= 0.17 p= 0.38 | rs= 0.01  p= 0.95 | rs= 0.22 p= 0.25 | rs= 0.44 p= 0.01 | rs= 0.28 p= 0.14 | rs =  -0.15  p= 0.43 | rs =  -0.25 p= 0.2. | rs =  -0.13 p= 0.49 |
| *bla*_OXA-2_ | rs =0.09 p=0.6184 | rs =0.11p=0.58 | rs =0.05p=0.79 | rs =0.09p=0.62 | rs =0.08 p=0.69 | rs =0.16 p=0.41 | rs = 0.56 p= 0.00 | rs =0.1 p=0.58 | rs =0.11 p=0.58 | rs =0.210 p=0.28 | rs =0.18 p=0.34 | rs =0.09 p=0.66 | rs =0.24 p=0.21 | rs =0.28 p=0.14 | rs =0.19 p=0.31 |
| *bla*_CMY-2_ | rs =0.67 p=0.0001 | rs =0.19 p=0.32 | rs =0.09 p=0.63 | rs =0.39 p=0.04 | rs =0.14 p=0.48 | rs =0.17 p=0.37 | rs =0.12 p=0.55 | rs =0.19 p=0.32 | rs =0.07 p=0.71 | rs =0.15 p=0.44 | rs =0.35 p=0.06 | rs =0.16 p=0.42 | rs =0.27 p=0.16 | rs =0.51  p=0.01 | rs =0.35 p=0.06 |
| *mcr*-1 | rs = 0.66 p= 8E-05. | rs= 0.19  p= 0.32 | rs= 0.09 p= 0.63 | rs= 0.10  p= 0.58 | rs= 0.14  p= 0.48 | rs= 0.17  p= 0.37 | rs =  -0.12  p= 0.55 | rs= 0.19 p= 0.32 | rs= 0.34  p= 0.07 | rs =  -0.08 p= 0.69 | rs =  -0.13 p= 0.52 | rs= 0.16 p= 0.42 | rs= 0.27 p= 0.16 | rs= 0.51 p= 0.01 | rs= 0.35p= 0.06 |

**Table S3**. Chi-square analysis of associations between serotypes and antimicrobial resistance genes

| Gene | Chi-square | Df | p-value | Adjusted p-value (FDR) | Significance |
| --- | --- | --- | --- | --- | --- |
| *cat*A1 | 6.55 | 7 | 0.477 | 0.625 | Not significant |
| *cml*A | 9.98 | 7 | 0.190 | 0.356 | Not significant |
| *flo*R | 7.20 | 7 | 0.409 | 0.625 | Not significant |
| *fos*A3 | 6.76 | 7 | 0.455 | 0.625 | Not significant |
| *tet*A | 11.34 | 7 | 0.125 | 0.268 | Not significant |
| *tet*B | 4.95 | 7 | 0.666 | 0.666 | Not significant |
| *qnr*A | 20.91 | 7 | 0.0039 | 0.0585 | Not significant |
| *ere*A | 5.30 | 7 | 0.624 | 0.666 | Not significant |
| *aac*(6′)-Ib-cr | 6.70 | 7 | 0.461 | 0.625 | Not significant |
| *aac*(3) | 6.20 | 7 | 0.516 | 0.625 | Not significant |
| *aad*A1 | 9.38 | 7 | 0.227 | 0.379 | Not significant |
| *aph*A1 | 12.71 | 7 | 0.079 | 0.198 | Not significant |
| *sul*I | 13.95 | 7 | 0.052 | 0.198 | Not significant |
| *sul*II | 10.23 | 7 | 0.176 | 0.356 | Not significant |
| *dfr*A1 | 13.58 | 7 | 0.059 | 0.198 | Not significant |

**Table S4.** Antimicrobial resistance, virulence, and biofilm profiles of *Salmonella* *enterica* isolates

| Isolate | Phenotypic antimicrobial resistance pattern | Genotypic Antimicrobial resistance pattern | Virulence gene | Biofilm gene |
| --- | --- | --- | --- | --- |
| S8: *S*. Colorado | C, AM, AX, SXT, CIP, NA, K, FF, DO, FOX, CTR, CAZ, AMC, ATM, FEP/CPM, E, S  MDRI=0.8  Type of resistance: XDR (Extensively drug-resistant) | *bla*_TEM_*, bla*_SHV_*, int*1  *cml*A*, flo*R*, tet*A*, qnr*A*, ere*A*, aad*A1*, aph*A*1, sul*I | *inv*A, *hil*A, *spi*C | *Csg*D |
| S7: *S*. Colorado | C, AM, AX, SXT, CIP, NA, K, FF, DO, FOX, CTR, CAZ, AMC, ATM, FEP/CPM, E, CN, S, APR  MDRI=0.9 - XDR | *bla*_TEM,_ *bla_SHV_, int*1  *fos*A3*, tet*A*, tet*B*, qnr*A*, ere*A*, aad*A1*, aph*A1*, sul*I*, sul*II | *inv*A, *hil*A, *stn*, *spi*C | *csg*D, *gcp*A |
| S9c: *S*. Kentucky | C, AM, AX, SXT, CIP, NA, K, FF, DO, FOX, CTR, CAZ, AMC, ATM, FEP/CPM, E, CN, S, APR  MDRI=0.9 - XDR | *bla*_TEM_*, bla*_CTX-M,_ *bla*_CMY-2_*, mcr-*1*, int*1*, cat*A1*, cml*A*, flo*R*, tet*A*, ere*A*, aac*(6′)-Ib-cr*, aph*A1*, sul*I*, sul*II*, dfr*A1 | *inv*A, *hil*A, *stn*, *spi*C | - |
| S9: *S*. Kentucky | C, AM, AX, SXT, CIP, NA, K, FF, DO, FOX, CTR, CAZ, AMC, ATM, FEP/CPM, E, CN, S, APR  MDRI=0.8 – XDR | *bla*_TEM_, *int*1, *cml*A, *flo*R, *tet*B, *aac* (3), *sul*I, | *inv*A, *hil*A, *stn* | *Csg*D |
| S15c: *S*. Jerusalem | C, AM, AX, SXT, CIP, NA, K, AK, FF, DO, FOX, CTR, CAZ, AMC, ATM, FEP/CPM, E, CN, S, APR  MDRI=0.9 – XDR | *bla*_TEM_, *bla*_SHV_, *bla*_CTX-M_  *mcr*-1, *int*1, *cat*A1, *cml*A, *flo*R, *tet*A, *tet*B, *ere*A,  *aac*(6′)-Ib-cr, *aad*A1, *aph*A1, *sul*I, *sul*II, *dfr*A1 | *inv*A, *hil*A, *stn*, *spi*C | *csg*D, *adr*A, *bcs*A |
| S12: *S*. Derby | C, AM, AX, SXT, CIP, NA, K, FF, DO, FOX, CTR, CAZ, AMC, ATM, FEP/CPM, E, S, APR  MDRI=0.8 – XDR | *bla*_TEM_*, int*1*, cat*A1*, cml*A*, flo*R*, tet*A*, tet*B*, , ere*A*, aac(3), aad*A1*, aph*A1*, sul*I*, sul*II*, dfr*A1 | *inv*A, *hil*A | *Csg*D |
| S18c: *S*. Kentucky | C, AM, AX, SXT, CIP, NA, K, FF, DO, FOX, CTR, CAZ, AMC, ATM, FEP/CPM, E, CN, S, APR  MDRI=0.8 - XDR | *tet*A, *aph*A1 | *inv*A, *hil*A, *stn* | *Csg*D |
| S18: *S*. Derby | C, AM, ATM, AMC, SXT, CIP, NA, AK, FF, FOX, CTR, CAZ, FEP/CPM, E, CN, S, APR  MDRI=0.8 – XDR | *int*1, *cml*A, *flo*R, *tet*A, *tet*B, *aph*A1 | *inv*A, *hil*A, *stn* | *Csg*D |
| S23: *S*. Kentucky | C, AM, AX, SXT, CIP, NA, K, FF, DO, FOX, CTR, CAZ, AMC, ATM, FEP/CPM, E, CN, S, APR  MDRI=0.8 - XDR | *bla*_TEM_, *bla*_CTX-M,_ *bla*_OXA-10_, int1, *cml*A, *flo*R, *tet*A, *tet*B, *ere*A, *aac*(6′)-Ib-cr, *aac*(3) ,*aad*A1, *aph*A1, *sul*I,  *dfr*A1 | *inv*A, *hil*A, *stn*, *spi*C | - |
| S22: *S*. Salamae | C, AM, AX, SXT, CIP, NA, K, FF, DO, FOX, CTR, CAZ, AMC, ATM, FEP/CPM, E, CN, S, APR  MDRI=0.9 – XDR | *bla*_TEM_, *bla*_OXA-10_, *int*1, *cml*A, *flo*R, *tet*A, , *ere*A, *aac*(3), *aad*A1, *aph*A1 | *inv*A, *hil*A | - |
| S20:  *S*. Derby | C, DO, AMC, NA, S, CIP, APR, AX, ATM, FF, AM, K, FOX, CTR, CAZ, E, SXT  MDRI=0.8 - XDR | *bla*_OXA-10_, *int*1, *cml*A, *flo*R, *tet*A, *ere*A ,*aad*A1, *aph*A1 | *inv*A, *hil*A, *spi*C, | *Csg*D |
| S19: *S*. Derby | C, AM, AX, SXT, CIP, NA, AK, K, FF, DO, FOX, CTR, CAZ, AMC, ATM, FEP/CPM, E, CN, S, APR  MDRI=0.9 - XDR | *bla*_TEM_, *bla*_OXA-10_  *int*1, *cml*A, *flo*R, *tet*A  *ere*A, *aad*A1, *aph*A1 | *inv*A, *hil*A, *stn*, *spi*C | - |
| S21: *S*. Salamae | C, DO, AX, K, AMC, NA, FF, CAZ, AM, SXT, FOX, ATM, E, CTR, FEP/CPM  MDRI=0.6 - MDR | *bla*_TEM_, *bla*_OXA-10_, *int*1, *cml*A, *flo*R, *tet*A, *ere*A, *aac*(6′ )-Ib-cr, *aac* (3) ,*aad*A1, *aph*A1, *sul*I | *inv*A, *hil*A, *stn*, *spi*C | *Csg*D |
| S19c: *S*. Derby | C, AM, AX, AMC, ATM, FOX, CTR, CAZ, FEP/CPM, FF, CIP, NA, E, S, K, SXT  MDRI=0.7  MDR (Multidrug-resistant) | *bla*_TEM_, *bla*_SHV_, *int*1, *cml*A, *flo*R, *tet*A, *ere*A, , *aph*A1 | *inv*A, *hil*A, *stn*, *spi*C | *Csg*D |
| S20c: *S*.Typhimurium | C, NA, APR, FOX, CIP, SXT, DO, K, FF, E, AMC, ATM, CAZ, S, CN, FEP/CPM  MDRI=0.7 – XDR | *bla*_TEM_, *bla*_OXA10_,  *int*1, *cml*A, *flo*R, *fos*A3, *tet*A, *tet*B, *aac*(3), *aph*A1 | *inv*A, *hil*A, *spi*C, | *Csg*D |
| S27 : *S*. Derby | C, AX, DO, AK, AMC, NA, FF, AM, SXT, CIP, FOX, ATM, CAZ, E, CTR, CN, K, FEP/CPM  MDRI=0.8 - XDR | *bla*_TEM_, *bla*_SHV_ *int*1, *cat*A1, *flo*R, *tet*A, *tet*B, *ere*A, *aac*(6′)-Ib-cr, *aph*A1, *sul*I, *dfr*A1 | *inv*A, *spi*C, *spv*C | *Csg*D |
| S21c: *S*. Salamae | C, NA, K, AMC, DO, FF, AX, SXT, AM, FOX, ATM, CAZ, E, CTR, CN, FEP/CPM  MDRI=0.7 – MDR | *bla*_TEM_, *bla*_OXA-10_, *int*1, *cml*A, *flo*R, *tet*A, *ere*A, *aad*A1, *aph*A1, *sul*I, *sul*II, *dfr*A1 | *inv*A, *hil*A, *stn*, *spi*C | - |
| S30: *S*. Kentucky | C, DO, APR, NA, AX, AMC, FF, SXT, AM, FOX, ATM, CAZ, E, CIP, CN, S, CTR, K, FEP/CPM  MDRI=0.9 – XDR | *bla*_TEM_, *bla*_OXA-10_, *int*1, *cml*A, *flo*R, *fos*A3, *tet*A, *tet*B, *ere*A, *aac*(3) ,*aad*A1, *aph*A1, *sul*I, *dfr*A1 | *inv*A, *hil*A, *stn*, *spi*C | *Csg*D |
| S31: *S*. Kentucky | C, DO, APR, NA, AX, AMC, FF, SXT, AM, FOX, ATM, CAZ, E, CIP, CN, S, CTR, AK, K, FEP/CPM  MDRI=0.8 – MDR | *bla*_TEM_, *bla*_CTX-M,_ *bla*_CMY-2_, *int*1, *cat*A1, *cml*A, *flo*R,  *fos*A3, *tet*A, *tet*B, *ere*A,, *aac*(3) , *aph*A1, *sul*I, *sul*II, *dfr*A1 | *inv*A, *hil*A, *stn*, *spi*C | *Csg*D |
| S29: *S*. Kentucky | C, NA, APR, AK, AMC, FF, AX, SXT, AM, FOX, ATM, CAZ, E, CIP, CN, S, CTR, K, FEP/CPM  MDRI=0.9 - XDR | *bla*_TEM_, *bla*_CTX-M_, *bla*_CMY-2_, *mcr*-1, *int*1, *cat*A1, *cml*A, ,*flo*R, *fos*A3, *tet*A, *tet*B, *ere*A, *aac* (3), *aph*A1, *sul*I, *sul*II, *dfr*A1 | *inv*A, *hil*A, *stn*, *spi*C | *csg*D, *adr*A |
| S28: *S*. Kentucky | C, NA, APR, K, AMC, FF, AX, SXT, AM, FOX, ATM, CAZ, E, CIP, CN, S, FEP/CPM  MDRI=0.7 - MDR | *bla*_TEM_, *bla*_SHV_, *int*1, *cml*A, *floR*, *fos*A3, *tet*A, *tet*B, *ere*A, *aac*(6′)-Ib-cr, *aac*(3), *aad*A1, *aph*A1, *sul*I, *dfr*A1 | *inv*A, *hil*A, *spv*C | *Csg*D |
| B17c: *S*. Infantis | C, NA, APR, AMC, FF, AX, S, AM, FOX, ATM, CAZ, E, CIP, CN, CTR, FEP/CPM  MDRI=0.7 – MDR | *bla*_TEM_, *bla*_SHV_, *int*1, *flo*R, *sul*I | *inv*A, *hil*A, *stn*, *spi*C | *csg*D, *adr*A |
| K2: *S*.Typhimurium | C, NA, APR, AK, AMC, FF, AX, SXT, AM, FOX, ATM, CAZ, E, CIP, CN, S, CTR, K, FEP/CPM, DO  MDRI=0.9 - XDR | *bla*_TEM_, *int*1, *cml*A, *flo*R, *tet*A, *ere*A, *sul*I, *sul*II, *dfr*A1 | *inv*A, *hil*A, *stn* | *Csg*D |
| B12: *S*. Infantis | C, NA, APR, AMC, FF, AX, SXT, AM, FOX, ATM, CAZ, E, CIP, CN, S, CTR, K, FEP/CPM, DO  MDRI=0.8 - XDR | *int*1, *flo*R, *tet*A, *tet*B, *ere*A, *aac* (3), *aph*A1, *sul*I | *inv*A, *hil*A, *stn*, *spi*C | *Csg*D |
| B35c: *S*. Kentucky | C, NA, APR, AMC, FF, AX, SXT, ATM, FOX, DO, CIP, CN, S, CTR, K, CAZ, FEP/CPM  MDRI=0.7- MDR | *bla*_TEM_, *int*1, *cml*A, *flo*R, *aad*A1, *aph*A1, *sul*I,  *dfr*A1 | *inv*A, *hil*A, *stn*, *spi*C | *Csg*D |
| B43: *S*.Typhimurium | C, NA, APR, AK, AMC, FF, AX, SXT, AM, FOX, ATM, CAZ, E, CIP, CN, S, CTR, K, FEP/CPM, DO  MDRI=0.8 – XDR | *bla*_TEM_, *bla*_CTXM_,  *int*1, *cat*A1, *flo*R, *tet*A, *tet*B, *ere*A, *aac*(6′ )-Ib-cr ,*aad*A1, *aph*A1, *sul*I | *inv*A, *hil*A, *stn*, *spi*C, *spv*C | *Csg*D |
| B44: *S*.Typhimurium | C, NA, APR, K, AMC, FF, AX, SXT, AM, FOX, ATM, CAZ, E, CIP, CN, S, CTR, IPM, FEP/CPM, DO  MDRI=0.9 - XDR | *bla*_CTX-M_, *bla*_OXA-2_, *int*1, *cml*A, *flo*R, *tet*A,  *qnr*A, *ere*A, *aac*(3),  *aad*A1, *aph*A1, *sul*II, *dfr*A1 | *inv*A, *hil*A, *spi*C | *Csg*D |
| B39: *S*. Kentucky | C, NA, APR, K, AMC, FF, AX, SXT, AM, FOX, ATM, CAZ, E, CIP, CTR, FEP/CPM, DO  MDRI=0.7 – XDR | *bla*_TEM_, *int*1, *cml*A, *flo*R, *tet*A, *aph*A1, *sul*I, *dfr*A1 | *inv*A, *hil*A, *stn*, *spi*C | *Csg*D |
| B19c: *S*.Virchow | C, NA, K, DO, AMC, FF, AX, SXT, AM, FOX, ATM, CAZ, E, CIP, CN, S, CTR, FEP/CPM  MDRI=0.8 – XDR | *bla*_TEM_, *int*1 , *cml*A, *flo*R, *ere*A, *aac* (3) | *inv*A, *hil*A, *stn* | *csg*D, *adr*A |
