# Supplementary material for: Genetic diversity and mobile genetic element associated multidrug resistance in Salmonella enterica from broiler chickens in Egypt
Source: Sci Rep. 2026 Jul 3;16:20548. doi: 10.1038/s41598-026-59913-w (PMC13332045; doi:10.1038/s41598-026-59913-w)
Supplement: Supplementary file 2 — Supplementary Material 2 [file 41598_2026_59913_MOESM2_ESM.docx]

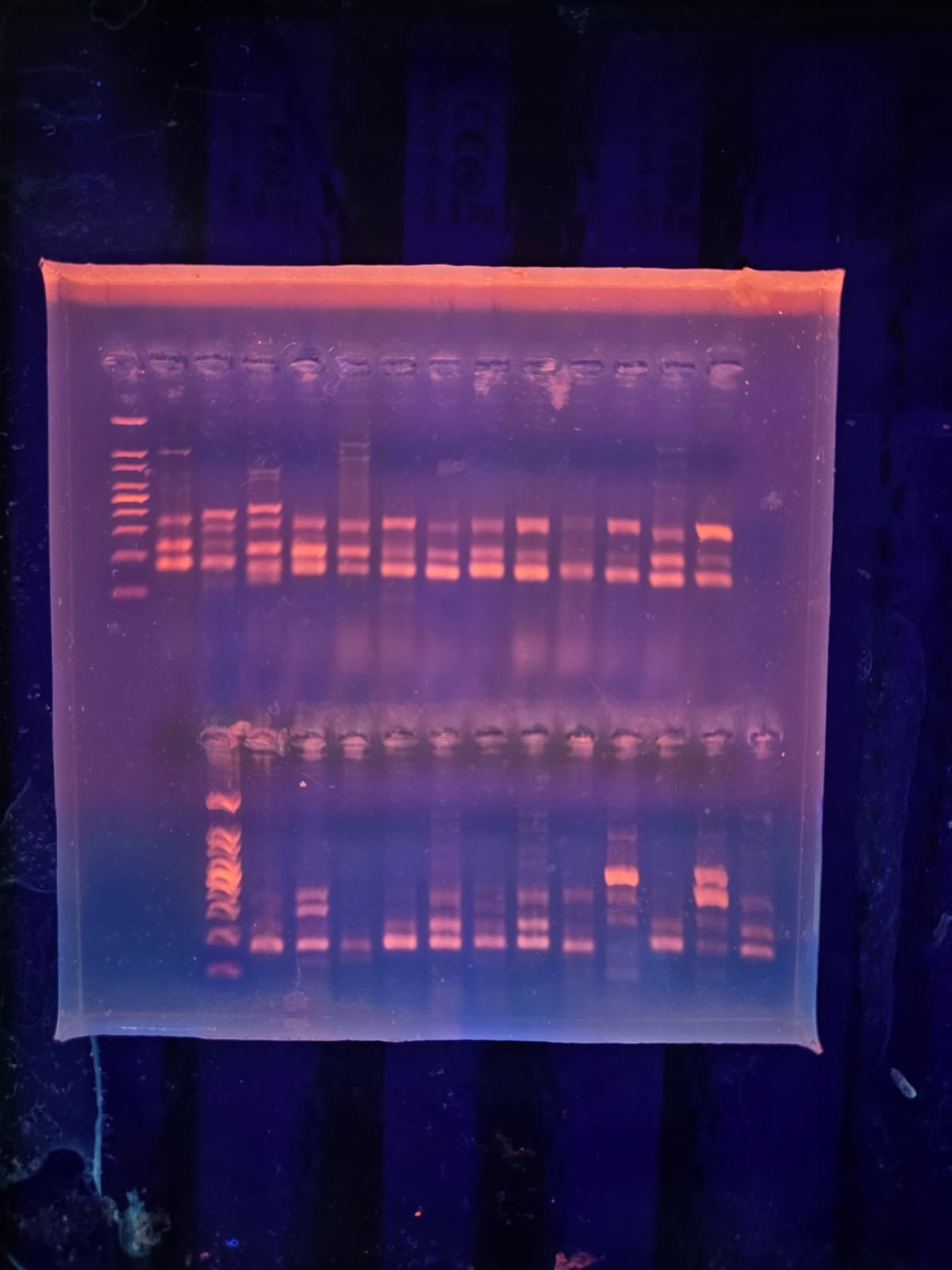


Full-length gel corresponding to Fig. 13. All PCR reactions were performed under identical experimental conditions.
